# Value-based decision making via sequential sampling with hierarchical competition and attentional modulation

**DOI:** 10.1371/journal.pone.0186822

**Published:** 2017-10-27

**Authors:** Jaron T. Colas

**Affiliations:** Computation and Neural Systems Program, California Institute of Technology, Pasadena, CA, United States of America; Centre national de la recherche scientifique, FRANCE

## Abstract

In principle, formal dynamical models of decision making hold the potential to represent fundamental computations underpinning value-based (i.e., preferential) decisions in addition to perceptual decisions. Sequential-sampling models such as the race model and the drift-diffusion model that are grounded in simplicity, analytical tractability, and optimality remain popular, but some of their more recent counterparts have instead been designed with an aim for more feasibility as architectures to be implemented by actual neural systems. Connectionist models are proposed herein at an intermediate level of analysis that bridges mental phenomena and underlying neurophysiological mechanisms. Several such models drawing elements from the established race, drift-diffusion, feedforward-inhibition, divisive-normalization, and competing-accumulator models were tested with respect to fitting empirical data from human participants making choices between foods on the basis of hedonic value rather than a traditional perceptual attribute. Even when considering performance at emulating behavior alone, more neurally plausible models were set apart from more normative race or drift-diffusion models both quantitatively and qualitatively despite remaining parsimonious. To best capture the paradigm, a novel six-parameter computational model was formulated with features including hierarchical levels of competition via mutual inhibition as well as a static approximation of attentional modulation, which promotes “winner-take-all” processing. Moreover, a meta-analysis encompassing several related experiments validated the robustness of model-predicted trends in humans’ value-based choices and concomitant reaction times. These findings have yet further implications for analysis of neurophysiological data in accordance with computational modeling, which is also discussed in this new light.

## Introduction

**Fig 1:**
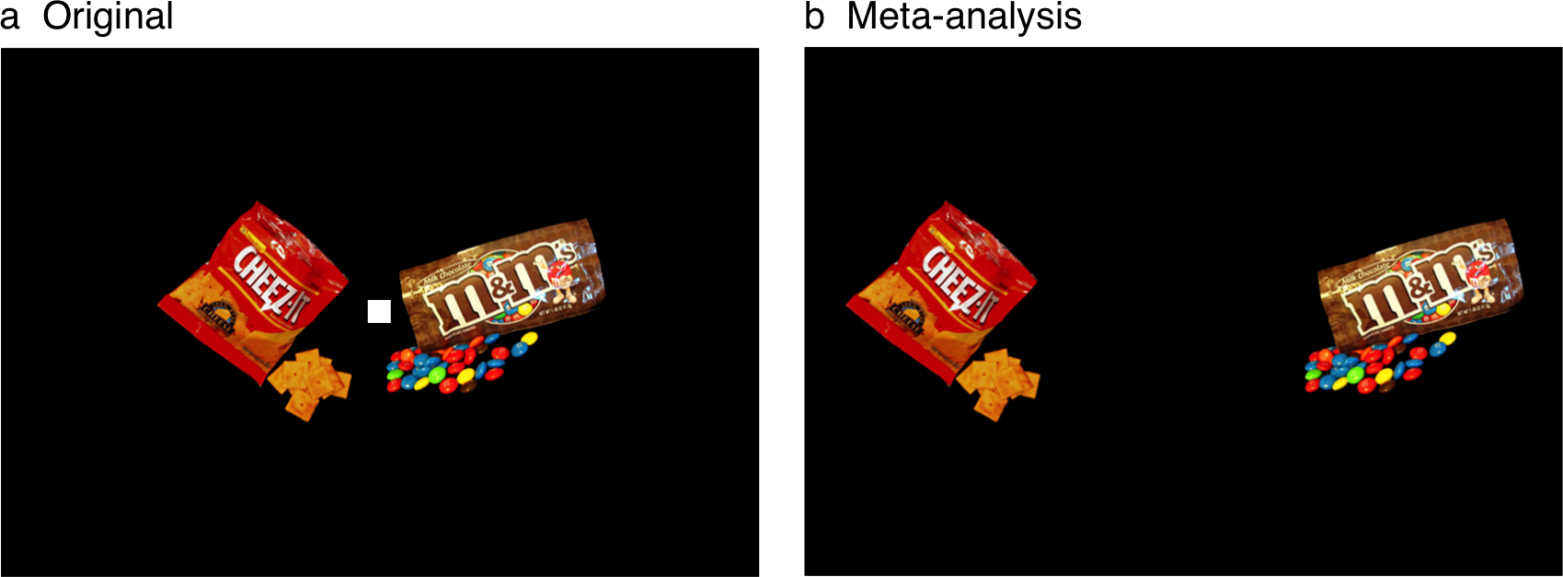
Task. **(a)** For all studies, subjects were required to make a two-alternative forced choice between a pair of randomly sampled foods with uncorrelated subjective values. The original data set to which the forthcoming computational models were fitted was distinguished by a paradigm with adjacent stimuli and persistent fixation, allowing for only covert shifting of the focus of visual attention. **(b)** In contrast, the other studies included in the meta-analysis featured stimuli that were well separated spatially and thus required eye movements.

How do we make value-based (i.e., preferential) decisions? A variety of computational models have put forth possible answers to this question in the form of general algorithms by which options are effectively compared and decided upon in the presence of noisy information [1]. With numerous existing models to choose among and so many possible models yet to be defined, the pressing key issues concerning which new models merit exploration and which models are best under which circumstances remain far from resolved. As theory ultimately must be reconciled with praxis and actual data, the present study took an empirical approach to model selection for a two-alternative forced-choice (2AFC) paradigm [2] involving the subjective values of foods (**Fig 1**).

Following the introduction of the sequential probability-ratio test (SPRT) [3–5], stochastic control theory offered an optimal standard [6] for dynamical modeling of decision-making processes and was adopted by cognitive psychology as the basis of the sequential-sampling models (SSMs) [7] that would rival the atemporal models of signal-detection theory [8]. Truest to the SPRT and since emerging as the most popular and influential SSM is the drift-diffusion model [7,9–11], which posits a unidimensional (or mirror-symmetric) process accumulating the relative evidence between alternatives (i.e., the log-likelihood ratio). An alternative to the drift-diffusion model commonly referred to as the race model [12–15] instead assumes a race of independent accumulators in parallel within a multidimensional system. In addition to boasting mathematical elegance, both of these models can be regarded as normative inasmuch as each adheres to a distinct definition of optimality (see Discussion).

Yet, recent advances in neuroscience have begun to lend insight toward a less prescriptive and more descriptive account of human decision making constrained by neural plausibility rather than simplicity, analytical tractability, or optimality. The implications of these advances are not limited to interpretation of neurophysiological signals. On the contrary, the present study reveals unique contributions of this neurocentric modeling to the emulation of human behavior. Measurements of the concomitant reaction time (RT) complemented measurements of discrete choices inasmuch as chronometry provides additional information for inference about neurophysiological and mental processes underlying behavior [16]. A substantial and growing body of theoretical and experimental work has solidified the notion that animals’ decisions are driven by diffusion-like sequential-sampling and integration-to-threshold processes in the nervous system [17,18]. That is, inputs in the form of reward-value or evidence signals are sampled and integrated into accumulating decision signals that activate respective execution signals upon reaching a threshold at which an action is selected. Rather than making decisions about the perceptual qualities of stimuli, subjects in the present study instead chose which of the two foods presented for each trial they would prefer to eat. Whereas research within this domain has typically emphasized the simpler case of perceptual decision making, more recent investigation has begun to suggest that such canonical computations are similarly implicated in value-based and economic decision making as well [19–21]. Invoking “field theory” [22,23] with its mathematical formalization of decision making in terms of topology, “decision field theory” [24,25] was among the first dynamical models to be explicitly related to preferential decisions, and SSMs originally intended for perceptual decision making were eventually suggested to generalize to other domains (e.g., [26]). Nevertheless, many questions remain as to pivotal details of the architectures of these putative dynamical systems, including the extent to which the representations of individual options interact [27–29].

Any computational model of decision making occupies a position along a spectrum [30] ranging from the most simple and abstract cognitive models to the most detailed and biophysically realistic models that explicitly represent properties of individual neurons and membrane proteins [31]. A connectionist model as desired here could stand as a middling hybrid to appease the tension between these dichotomous extremes, each of which entail advantages and disadvantages with respect to accuracy, parsimony, and interpretability. The present work implicitly tested for oft-overlooked modulatory effects of attention [32,33] and its associated positive-feedback loops as well as essential aspects of established neuroalgorithmic models—namely, the feedforward-inhibition model [34,35], the leaky-competing-accumulator (LCA) model [26,36], and the divisive-normalization model [37–40], which actually has origins outside the realm of SSMs. Prior studies have generally evaluated SSMs using stimuli that vary along a single dimension and are thus intrinsically competitive, such as in a signal-detection or motion-discrimination task. Crucially, the 2AFC paradigm explored herein is distinguished by alternatives with parameters that are statistically independent across trials [29,41]. This feature enabled rigorous assessment of competitive mechanisms or lack thereof.

In the spirit of Occam’s razor and the proverbial assertion that “all models are wrong, but some are useful” [42], various dynamical models were compared with an aim for achieving an ideal balance of parsimony and accuracy [43], where the latter reflects both empirical fitting performance and theoretical neural plausibility. Temporality was essential, as effects on observed RT—that is, half of the available behavioral data—are beyond the scope of any static model. Moreover, applicability to computational-model-based analysis of neurophysiological data [44,45] imposed additional constraints. A novel synthesis of key concepts at a moderate level of complexity was to quantitatively account for this class of value-based decisions in a sizeable data set including RT distributions from human subjects. Furthermore, a meta-analysis of experiments similarly involving binary choices about randomly sampled foods with uncorrelated values went on to reveal qualitative trends across multiple independent data sets that could be related to predictions of this novel hybrid model.

## Materials and methods

### Participants

Participants in all of the individual studies were generally healthy volunteers between 18 and 40 years old from Caltech and the local community. The number of participants included in each study is listed in **Table 1**. Participants in the JC1, JC2, and SL studies were all right-handed. Across all studies, criteria for participation included enjoying and regularly eating common American snack foods such as those used for the experiments. Participants provided informed written consent for every individual study’s protocols, which were in this and all other cases approved by the California Institute of Technology Institutional Review Board. Participants were paid for completing a study and always received a chosen food item.

**Table 1:** Meta-analysis: Data sets.

Data set	Subjects	Trials	Values	Details
J. Colas 1 (JC1)	31	21,394	4	fixation, 3 cond. (actions), EEG
J. Colas 2 (JC2)	27	9,174	4	3 cond. (actions), fMRI
C. Hutcherson (CH)	34	1,632	5	mouse, control condition only
I. Krajbich, 2010 (IK)	39	3,791	11	
S. Lim (SL)	24	8,549	7	2 cond. (approach/avoid), fMRI
Colas & J. Lu, 2017 (JL)	35	13,992	5	4 cond. (spatial bias)
N. Sullivan, 2015 (NS)	28	5,560	5	mouse, health-conscious
Aggregate	218*	64,092		

Listed for each of the studies included in the meta-analysis are the number of subjects, the number of trials across subjects, the number of discrete option values that were to be normalized to a common range prior to analysis, and miscellaneous notable details.

*This total does not account for subjects who participated in more than one study.

### Experimental procedures: Modeled data set

Prior to acquisition of the “JC1” data set proper, the subject first completed an ancillary rating task that solicited the subjective values of all stimuli with linear rankings. Images of 70 generally appetitive snack foods were presented against a black background one at a time. The subject reported the desirability of eating each food at the end of the experiment according to a 5-point scale (0: “not at all”, 1: “slightly”, 2: “moderately”, 3: “strongly”, 4: “extremely”). The subject was given unlimited time to respond by pressing one of five buttons along a row on a keyboard with the right hand. As feedback, the selected rating was presented centrally as a white Arabic numeral during an intertrial interval of 1000 ms. The orientation of the scale was counterbalanced across subjects so that neither side was consistently associated with positive valence. The order of stimulus presentation was randomized for each subject. These images were chromatic and had a resolution of 288 x 288 pixels and each subtended 8.0° x 8.0° of visual angle. Stimuli were presented on a 23-inch LCD monitor with a resolution of 1024 x 768 pixels from a distance of 100 cm as part of an interface programmed using MATLAB and the Psychophysics Toolbox [46].

Stimuli were randomly selected to form 720 pairs for the subject’s unique sequence of trials in the main choice task (i.e., for the modeled JC1 data set) as follows. Only foods with a rating of subjective value greater than zero were included. Pairs were first selected so as to balance the differences in value ranging from 0 to 3 as much as possible. Each pair of values among the ten possible combinations was also balanced within each value-difference bin. The side on which the food with greater value was presented was counterbalanced within each of the ten combinations. Stimuli were never repeated in consecutive trials.

The subject was allotted 3 s to choose between a pair of food stimuli presented adjacently to each other on either side of the white fixation spot (**Fig 1A**). Incidentally, electroencephalography (EEG) data were also being acquired while the subject performed this choice task. Thus, the subject needed to maintain fixation at all times during trials to prevent eye-movement artifacts from contaminating EEG signals. This task also featured three main experimental conditions in randomly ordered blocks of 60 trials with balanced values: the subject would choose by pressing one of two buttons with either index finger, by stepping on one of two pedals, or with the actions unknown until the time of choosing is indicated. Whereas the subject immediately indicated the choice using the appropriate action for the button and pedal conditions, the unknown condition instead required that the time of choice first be indicated without regard to action by pressing the space bar with the right thumb. This nonspecific response, which corresponded to the relevant reaction time (RT), would initiate a cue in the form of the letter H above fixation or the letter F below fixation as instruction for a button or pedal response, respectively. Only 800 ms was allotted to subsequently indicate which item was chosen in the unknown condition’s second phase so as to prevent further deliberation after reporting that a decision had been made. Thus, the data from all three conditions could be concatenated prior to analysis. The subject was prepared for the time constraint of the unknown condition with practice trials as well as at least 100 trials of a task with the same timing that merely required reporting which randomly selected side of the screen a gray square appeared on for each trial. The action cues of the unknown condition were randomly counterbalanced for each subject. These cues were colored cyan and yellow with the color mapping counterbalanced across subjects. Trials were separated by an intertrial interval drawn from a uniform distribution ranging between 2500 and 3500 ms, and self-paced breaks for blinking and other movements that must be restricted for EEG were available every three trials.

The subject was required to refrain from eating or drinking anything except for water for at least 2 hours prior to the start of the experiment. The procedure was incentive-compatible [47] inasmuch as the hungry subject was informed that one of the choices made was to be selected randomly and implemented at the end of the session. That is, upon completion, the subject was provided with this chosen food and required to consume it. Failure to choose in time for any trial resulted in the choice being made randomly by the computer, such that the subject could not avoid any choice.

### Experimental procedures: Meta-analysis

The meta-analysis included six additional data sets (**Table 1**). Common to these studies was the basic scheme of a 2AFC task for which subjects made incentive-compatible preferential decisions about randomly sampled foods with values that were uncorrelated across trials; however, unlike the original (i.e., JC1) study that was modeled, the stimuli were always presented separately on opposite sides of the display with no restrictions on eye movements (**Fig 1B**). Option values were similarly derived from single-stimulus rating tasks, and the number of possible values is listed for each study in **Table 1**. The specific details of the experimental procedures of these studies are not directly relevant to the meta-analysis, but their primary distinguishing features are described here.

The “JC2” data set was taken from a functional magnetic-resonance imaging (fMRI) study analogous to the original EEG study. As mentioned previously, however, eye movements were allowed. Moreover, the subject was instead allotted 4560 ms to respond.

The “CH” data set was taken from the blocked control condition of a mouse-tracking study. In the two experimental conditions omitted here, decisions were not made naturally but rather on the basis of either only taste or only healthiness. Instead of responding with a conventional button press, the subject used a computer mouse to move a cursor from the center of the bottom of the display to the location of the preferred food in either the upper-left or the upper-right corner and clicked within a rectangle surrounding the image. This mouse-click response was delivered within 4 s.

The “IK” data set [33] was taken from an eye-tracking study with the most standard version of the 2AFC task. The subject was given unlimited time to respond.

The “SL” data set was taken from an fMRI study including two experimental conditions that were collapsed prior to analysis, as with the JC1 and JC2 data sets. This study was unique in that generally aversive foods were also included in equal proportion in the set of stimuli. Seven possible values emerged from averaging of two separate ratings along a 4-point scale. Whereas the subject simply indicated the preferred food in the “approach” condition, the instruction was to instead indicate the nonpreferred food in the “avoid” condition. The subject was allotted 3 s to respond.

The “JL” data set [48] was taken from an eye-tracking study including four between-subject experimental conditions divided into two blocks of trials each that could all be analyzed together. The essential manipulation was that for one of the two blocks the stimulus with greater value was presented on the same side of the display for 90% of the trials. The four conditions corresponded to a control block followed by a leftward-bias block, a control block followed by a rightward-bias block, a leftward-bias block followed by a control block, and a rightward-bias block followed by a control block. The relatively subtle effects of the learned spatial biases could be averaged out for the sake of simplicity. The subject was given unlimited time to respond.

The “NS” data set [49] was taken from a second mouse-tracking study. Although the instruction was simply to choose the more desirable food, the subject was also reminded to be health-conscious with the presentation of information concerning the importance of healthy eating before the task. The subject was given unlimited time to respond.

### Computational modeling

The neural-network framework common to all of the models posits that separate populations of neurons represent the decision signals specific to each option under consideration. These neuronal ensembles are reduced to individual units in a connectionist scheme, such that the decision signal *d*_*x*_*(t)* corresponds to the current aggregate level of activity in the decision-making neurons representing alternative *x* at time *t*. These decision signals are initialized to zero at stimulus onset (i.e., *t* = 0) as follows:

$$\forall \;x:\;d_{x}(t=0)=0$$

The latent value *V*_*x*_ of each alternative is unknown at stimulus onset, as the processes underlying stimulus recognition and evaluation require some time. Thus, the value signal *v*_*x*_*(t)* within an ensemble of value-encoding neurons is initialized to zero and subsequently elevates to *V*_*x*_ as a step function after the constant predecision time *T*_*0*_ has elapsed like so:

$$\forall hx:\;v_{x}(t)=\left\{ \begin{array}{l} 0,\;t<T_{0}\\ V_{x},\;t\geq T_{0} \end{array}\right.$$

The fixed predecision time for the value-signal input was biologically constrained to be 150 ms for this paradigm (see Discussion). Time is discretized here to reflect the iterative implementations of these algorithms in practice as approximations of differential equations in continuous time. While every decision signal remains below the threshold level *D* (an arbitrary positive constant here set to 100 to represent 100%), the Markov process evolves by fixed time increments Δ*t* (here set to 10 ms) according to this generalized recurrence relation:

$$d_{x}(t+\Delta t)=\max\left\{0,d_{x}(t)+f_{x}(t)+\varepsilon_{x}(t)\right\}$$

**Fig 2:**
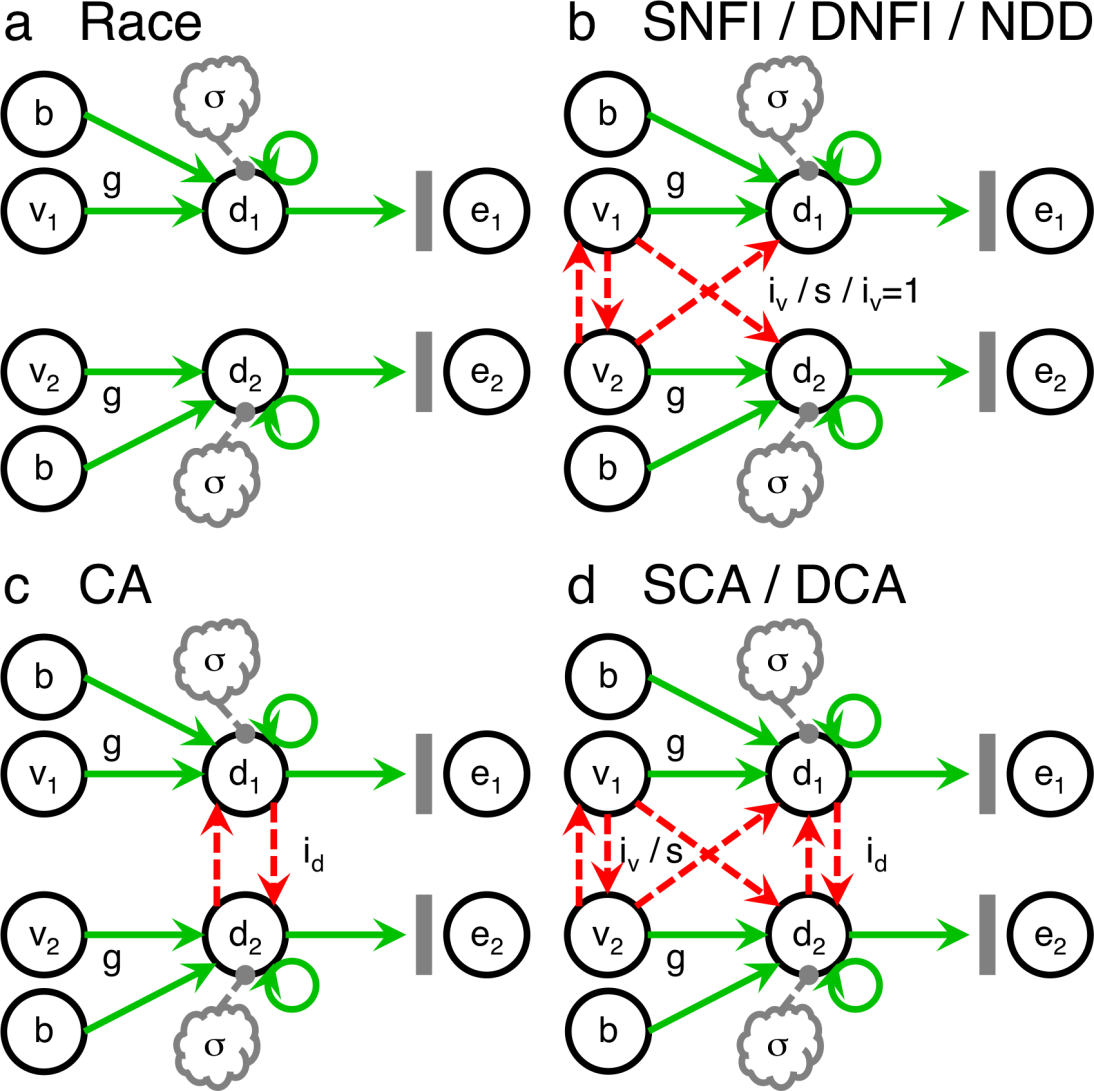
Dynamical models of neural decision making. **(a)** The race model [12–15] is the most basic of these by virtue of assuming that the representations of each option are completely independent. **(b)** Input-dependent competition is the signature feature common to the subtractive normalization-or-feedforward-inhibition (SNFI) model [34,35], the divisive normalization-or-feedforward-inhibition (DNFI) model [37–40], and the neural drift-diffusion (NDD) model [7,9–11]. The NDD model is nested within the SNFI model but instead posits perfect competition (i.e., *i*_*v*_ = 1). **(c)** The competing-accumulator (CA) model [26,36] is instead characterized by state-dependent competition via lateral inhibition at the level of accumulating decision signals. **(d)** The subtractive competing-accumulator (SCA) and divisive competing-accumulator (DCA) models take a novel approach of including both input-dependent competition and state-dependent competition in tandem. Solid green and dashed red arrows indicate excitatory and inhibitory connections, respectively. At the level of value signals, the leftmost vertical and diagonal dashed red arrows denote lateral inhibition (i.e., input normalization or relative coding) and feedforward inhibition, respectively, which are represented collectively here because in this context they are equivalent in terms of output. The gray clouds reflect independent sources of noise. Vertical gray bars symbolize thresholding mechanisms. *v*_*x*_ represents the ensemble of value-encoding neurons representing alternative *x*. *d*_*x*_ represents the corresponding ensemble of decision-making neurons. *e*_*x*_ represents the corresponding ensemble of execution neurons. The free parameters are *b* for baseline input, *g* for the gain of value-signal inputs, σ for noise, *i*_*v*_ for value-signal inhibition as part of a subtractive transformation, s for semisaturation as part of a divisive transformation, and *i*_*d*_ for decision-signal inhibition.

**Fig 3:**
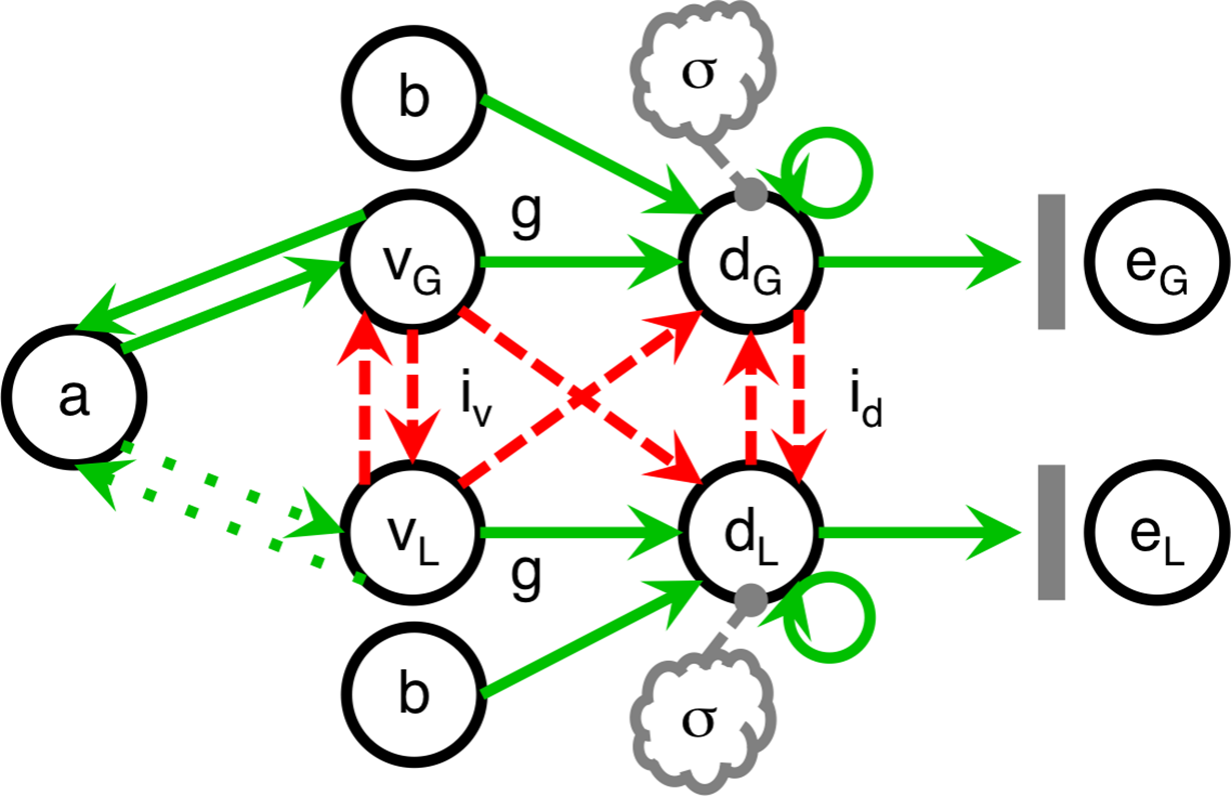
The supralinear subtractive competing-accumulator (SSCA) model. The SSCA model builds upon the SCA model with the intention of approximating the net effects of the addition of an attentional module that selectively modulates value signals. The positive-feedback loops that are consequently formed generate disproportionate amplification of value signals that are already greater in magnitude, thus promoting “winner-take-all” processing [32]. This schematic only depicts a positive-feedback loop at the level of value signals to adhere more closely to the parsimonious implementation used here with a static supralinear power law requiring only one free parameter, *a*. However, also plausible are loops at the next level bridging decision-making signals and attentional processes either with or without intermediate value signals. The contrast between solid and dotted green lines symbolizes the asymmetry in the positive-feedback loop’s impact on each alternative’s representation. As time progresses, there is an increasingly higher probability of attention being directed at the alternative with greater value, which is denoted by the *G* subscript, rather than the alternative with lesser value, which is denoted by the *L* subscript.

The first decision signal to reach the threshold level of activity *D* immediately triggers the respective execution signal *e*_*x*_*(t)* for the alternative represented. This motoric execution signal takes the form of a step function that defines the RT upon onset and also resets the entire system in preparation for the next trial. A threshold-linear activation function is implemented with the max operator to rectify negative activity, which is neurally implausible and also would exaggerate the effects of lateral inhibition if present. The first recursive term, *d*_*x*_*(t)*, produces perfect integration across time by means of balanced recurrent self-excitation and leakage. The final term, *ε*_*x*_*(t)* (or *N(0σ*,^*2*^*)*_*x*_*(t)* henceforth to be explicit), combines all sources of noise into a Gaussian distribution with mean μ = 0 and parameterized standard deviation *σ* that is drawn from independently within each alternative’s subsystem at every time step. The middle term, *f*_*x*_*(t)*, collectively represents all of the terms that vary across the individual models compared (**Figs 2 & 3**, **Table 2**).

**Table 2:** Model parameters.

Model	df	Baseline (*b*) Gain (*g*) Noise (σ)	Input-dependent competition (*i*_*v*_ or *s* or *i*_*v*_ *= 1*)	State-dependent competition (*i*_*d*_)	Power law as attention (*a*)
Race	3	Free	Absent	Absent	Absent
NDD	3	Free	Fixed / Subtractive (1)	Absent	Absent
SNFI	4	Free	Free / Subtractive (*i*_*v*_)	Absent	Absent
DNFI	4	Free	Free / Divisive (*s*)	Absent	Absent
CA	4	Free	Absent	Free	Absent
SCA	5	Free	Free / Subtractive (*i*_*v*_)	Free	Absent
DCA	5	Free	Free / Divisive (*s*)	Free	Absent
SSCA	6	Free	Free / Subtractive (*i*_*v*_)	Free	Free

All of the candidate models share three free parameters that correspond to baseline input (*b*), gain (*g*), and noise (σ), but the former two take on a different form in the divisive models. The SNFI and DNFI models introduce an additional free parameter for subtractive (*i*_*v*_) or divisive (*s*) input-dependent competition, respectively. Nested within the SNFI model is the NDD model for *i*_*v*_ = 1. The CA model instead introduces a free parameter for state-dependent competition (*i*_*d*_). The SCA and DCA models combine the CA model with the SNFI and DNFI models, respectively. The SSCA model adds a sixth free parameter (*a*) for a static supralinear power law approximating attentional modulation. The models are listed in ascending order of complexity. Divisive models are recognized as being inherently more complex than their subtractive counterparts irrespectively of degrees of freedom. Additionally, state-dependent competition is recognized as being inherently more complex than input-dependent competition. “df” stands for degrees of freedom.

### The race model

The race model (**Fig 2A**) [12–15] postulates the most basic of the algorithms tested with complete independence at all levels of the process. Thus, the recurrence relation for the decision signal is only modified as follows:

$$d_{x}(t+\Delta t)=\underset{\;}{\max}\left\{0,d_{x}(t)+b+gv_{x}(t)+{N(0,\sigma^{2})}_{x}(t)\right\}$$

The positive constant *b* corresponds to the baseline input (e.g., urgency signals) common to every ensemble of decision-making neurons. The positive constant *g* represents the gain of the value-signal input *v*_*x*_*(t)*.

### The neural drift-diffusion (NDD) model

The standard drift-diffusion model [7,9–11] is neurally implausible to the extent that it is unidimensional, which would translate to negative activation as the signal is biased toward an arbitrarily designated alternative. A two-channel representation of the standard drift-diffusion model can always be reduced to a single dimension because the mirror-symmetric paired signals are perfectly anticorrelated by definition and lack independent sources of noise. Thus, a neural drift-diffusion (NDD) model (**Fig 2B**) was contrived to be relatable to the other models within this neural-network framework. This similarity was to facilitate comparison and emphasize specifically the ramifications of perfect competition between inputs. That is, this neural implementation still retains the distinguishing feature of sensitivity to differences in input alone, as reflected here (where *n* denotes the number of alternatives):

$$d_{x}(t+\Delta t)=\underset{\;}{\max}\left\{0,d_{x}(t)+b+g(v_{x}(t)-\frac{1}{n-1}\sum_{y\neq x}v_{y}(t))+{N(0,\sigma^{2})}_{x}(t)\right\}$$

This parsimonious “max-minus-average” variant of the drift-diffusion model extended to multiple alternatives could be regarded as less optimal than the “max-minus-next” variant with a drift rate that only reflects the difference between the two signals with greatest magnitude by means of an obscure filtering process (see Discussion). Nevertheless, this distinction becomes irrelevant in the present case of two alternatives (i.e., *n* = 2), which reduces the general equation for alternative *x* to the following pair of equations:

$$d_{1}(t+\Delta t)=\underset{\;}{\max}\left\{0,d_{1}(t)+b+g(v_{1}(t)-v_{2}(t))+{N(0,\sigma^{2})}_{1}(t)\right\}$$

$$d_{2}(t+\Delta t)=\underset{\;}{\max}\left\{0,d_{2}(t)+b+g(v_{2}(t)-v_{1}(t))+{N(0,\sigma^{2})}_{2}(t)\right\}$$

### The subtractive normalization-or-feedforward-inhibition (SNFI) model

The subtractive normalization-or-feedforward-inhibition (SNFI) model (**Fig 2B**) [34,35] resembles the NDD model with a similar subtractive term but also adds a free parameter to render that input-dependent competition imperfect like so:

$$d_{x}(t+\Delta t)=\underset{\;}{\max}\left\{0,d_{x}(t)+b+g(v_{x}(t)-i_{v}\sum_{y\neq x}v_{y}(t))+{N(0,\sigma^{2})}_{x}(t)\right\}$$

For two alternatives, the equation is again reducible to a simpler pair of equations:

$$d_{1}(t+\Delta t)=\underset{\;}{\max}\left\{0,d_{1}(t)+b+g(v_{1}(t)-i_{v}v_{2}(t))+{N(0,\sigma^{2})}_{1}(t)\right\}$$

$$d_{2}(t+\Delta t)=\underset{\;}{\max}\left\{0,d_{2}(t)+b+g(v_{2}(t)-i_{v}v_{1}(t))+{N(0,\sigma^{2})}_{2}(t)\right\}$$

The NDD model is thus a special case of the SNFI model where *i*_*v*_ = 1/(*n*-1), such that *i*_*v*_ = 1 for *n* = 2. The constant *i*_*v*_ (with the constraint 0 ≤ *i*_*v*_ ≤ 1) represents value-signal inhibition ambiguously and potentially corresponds to the combined influence of lateral inhibition (i.e., input normalization or relative coding as opposed to absolute coding) and feedforward inhibition. To be precise, lateral inhibition should actually be incorporated into an equation representing the value-signal input *v*_*x*_*(t)*, whereas feedforward inhibition would remain as is in the equation for decision signals. This distinction is relevant for actual nervous systems. At this level of abstraction, however, lateral and feedforward inhibition are represented collectively in simplified equations because the two variants are ultimately mathematically equivalent insofar as each can mimic the other at the levels of decision signals and behavioral output.

### The divisive normalization-or-feedforward-inhibition (DNFI) model

The divisive normalization-or-feedforward-inhibition (DNFI) model (**Fig 2B**) [37–40] is merely the divisive analog of the SNFI model with the recurrence relation modified as follows:

$$d_{x}(t+\Delta t)=max\left\{0,\;{\;d}_{x}(t)+g\frac{b+v_{x}(t)}{s+\sum_{y}v_{y}(t)}+{N(0,\sigma^{2})}_{x}(t)\right\}$$

For two alternatives, this translates to the following reduction:

$$d_{1}(t+\Delta t)=max\left\{0,\;{\;d}_{1}(t)+g\frac{b+v_{1}(t)}{s+v_{1}(t)+v_{2}(t)}+{N(0,\sigma^{2})}_{1}(t)\right\}$$

$$d_{2}(t+\Delta t)=max\left\{0,\;{\;d}_{2}(t)+g\frac{b+v_{2}(t)}{s+v_{1}(t)+v_{2}(t)}+{N(0,\sigma^{2})}_{2}(t)\right\}$$

The positive constant *s* denotes semisaturation. As was also the case for the SNFI model, the simplified notational convention places input-dependent competition entirely within the equation for the decision signal rather than that for the value signal despite the ambiguity between lateral and feedforward inhibition at the level of value signals. Even without a quantifiable confound in degrees of freedom, the divisive transformation entails a less parsimonious assumption than a subtractive transformation by virtue of the complexity inherent to an actual neural implementation of shunting inhibition or otherwise divisive inhibition [50–52]. Another consideration—one that is also relevant for other computational mechanisms explored herein—is that the divisive transformation itself could emerge from a process with more temporally complex properties [53]. However, the simpler model of divisive normalization from which the DNFI model is derived has in fact been suggested to account for empirically observed neuronal activity thought to encode motivational value [38].

### The competing-accumulator (CA) model

The competing-accumulator (CA) model (**Fig 2C**) [26,36] substitutes state-dependent competition (i.e., dependent on the state of a decision signal) in lieu of input-dependent competition as the means by which each alternative’s representations interact, producing a more complex recurrence relation:

$$d_{x}(t+\Delta t)=\underset{\;}{\max}\left\{0,d_{x}(t)+b+gv_{x}(t)-i_{d}\sum_{y\neq x}d_{y}(t)+{N(0,\sigma^{2})}_{x}(t)\right\}$$

For two alternatives, the system is described by these coupled equations:

$$d_{1}(t+\Delta t)=\underset{\;}{\max}\left\{0,d_{1}(t)+b+gv_{1}(t)-i_{d}d_{2}(t)+{N(0,\sigma^{2})}_{1}(t)\right\}$$

$$d_{2}(t+\Delta t)=\underset{\;}{\max}\left\{0,d_{2}(t)+b+gv_{2}(t)-i_{d}d_{1}(t)+{N(0,\sigma^{2})}_{2}(t)\right\}$$

The constant *i*_*d*_ (with the constraint 0 ≤ *i*_*d*_ ≤ 1) represents decision-signal inhibition, which only reflects the lateral inhibition between competing ensembles of decision-making neurons.

### The subtractive competing-accumulator (SCA) model

The subtractive competing-accumulator (SCA) model (**Fig 2D**) synthesizes the SNFI and CA models with subtractive input-dependent competition and subtractive state-dependent competition acting in concert as written here:

$$d_{x}(t+\Delta t)=\underset{\;}{\max}\left\{0,d_{x}(t)+b+g(v_{x}(t)-i_{v}\sum_{y\neq x}v_{y}(t))-i_{d}\sum_{y\neq x}d_{y}(t)+{N(0,\sigma^{2})}_{x}(t)\right\}$$

For two alternatives, the same reductions apply to produce the following coupled equations:

$$d_{1}(t+\Delta t)=\underset{\;}{\max}\left\{0,d_{1}(t)+b+g(v_{1}(t)-i_{v}v_{2}(t))-i_{d}d_{2}(t)+{N(0,\sigma^{2})}_{1}(t)\right\}$$

$$d_{2}(t+\Delta t)=\underset{\;}{\max}\left\{0,d_{2}(t)+b+g(v_{2}(t)-i_{v}v_{1}(t))-i_{d}d_{1}(t)+{N(0,\sigma^{2})}_{2}(t)\right\}$$

### The divisive competing-accumulator (DCA) model

The divisive competing-accumulator (DCA) model (**Fig 2D**) is the divisive analog of the SCA model and instead synthesizes the DNFI and CA models with divisive input-dependent competition and subtractive state-dependent competition per the following algorithm:

$$d_{x}(t+\Delta t)=max\left\{0,\;{\;d}_{x}(t)+g\frac{b+v_{x}(t)}{s+\sum_{y}v_{y}(t)}-i_{d}\sum_{y\neq x}d_{y}(t)+{N(0,\sigma^{2})}_{x}(t)\right\}$$

For two alternatives, this can again be reduced to a pair of coupled equations:

$$d_{1}(t+\Delta t)=max\left\{0,\;{\;d}_{1}(t)+g\frac{b+v_{1}(t)}{s+v_{1}(t)+v_{2}(t)}-i_{d}d_{2}(t)+{N(0,\sigma^{2})}_{1}(t)\right\}$$

$$d_{2}(t+\Delta t)=max\left\{0,\;{\;d}_{2}(t)+g\frac{b+v_{2}(t)}{s+v_{1}(t)+v_{2}(t)}-i_{d}d_{1}(t)+{N(0,\sigma^{2})}_{2}(t)\right\}$$

### The supralinear subtractive competing-accumulator (SSCA) model

The supralinear subtractive competing-accumulator model (SSCA) model (**Fig 3**) retains all of the features of the best-performing SCA model with only one exception to relate to the concept of attentional modulation. Rather than being encoded in a linear fashion, value signals are transformed according to a power law determined by the constant *a* (with the constraint *a* ≥ 1) as the exponent. As a static approximation of dynamic processes, this strictly supralinear exponentiation is intended to capture the net effects of attention, which tends to be drawn to the representations of options with greater value and thus selectively amplifies them as part of a positive-feedback loop promoting “winner-take-all” processing [32] (see Discussion). Whereas the recurrence relation for the decision signal of the SCA model remains unchanged, the value signal is instead modeled with this new equation:

$$\forall sx:\;v_{x}(t)=\left\{ \begin{array}{l} 0,\;t<T_{0}\\ V_{x}^{a},\;t\geq T_{0} \end{array}\right.$$

### Model fitting

The free parameters of each model (**Table 2**) were fitted to the original JC1 data set using a standard chi-squared fitting method as follows [54]. Trials were first arbitrarily divided between training and test data sets of equal size according to the parity of the trials’ indices; odd-numbered trials from odd-numbered subjects and even-numbered trials from even-numbered subjects were assigned to training, and the remaining half of the trials were reserved for subsequent out-of-sample validation. Excessively fast contaminant observations (only 8 in total) were omitted below a lower limit of 300 ms, which accounts for the cumulative temporal constraints of visual recognition, decision making, and motoric execution. Data were concatenated across experimental conditions and subjects to sample RT distributions sufficiently and compensate for having few trials per subject and infrequent incorrect responses. Taking only the training data, the frequencies of either choice and the 10, 30, 50, 70, and 90% quantiles (i.e., six bins) of their respective RT distributions were calculated for each of the ten possible input vectors pairing the four linearly ranked input values. These input vectors were assigned equal weight in fitting to capture parametric effects. For comparison, Monte Carlo simulation was employed to generate 2,000 trials with each input vector for a given model and a given set of parameters. A χ^*2*^ statistic served as the objective function to be minimized, and the tuning parameters were optimized with respect to goodness of fit using iterations of the Nelder-Mead simplex algorithm [55] with randomized seeding.

In addition to the generative models, two discriminative models were fitted to the data to provide extreme upper and lower benchmarks for fitting performance. The saturated model was used to predict behavior in the test data using all of the training data directly, thus maximizing the degrees of freedom in accordance with the number of observations. The null model with a minimal three degrees of freedom assumes no effects of different inputs; rather, the mean choice frequencies across inputs were extracted along with the means of the minima and maxima of the RT distributions across both inputs and choices to define the range of a single uniform distribution for prediction.

Comparing models in a pairwise manner, likelihood-ratio tests were first used to verify the statistical significance of any improvement in fitting performance. Moreover, for the model comparison as a whole, penalties were imposed for model complexity at two standard levels using either the Akaike information criterion with correction for finite sample size (AICc) [56,57] or the stricter Bayesian information criterion (BIC) [58].

### Data analysis

The best-fitting instantiations of the models were used to simulate 20,000 trials with each of the ten possible input vectors. Trials were first classified into three distinct categories within the empirical data set and the simulated data set for juxtaposition as follows. Correct choices consistent with value ratings occurred when the option with greater value was chosen. Incorrect choices not consistent with value ratings occurred when the option with lesser value was chosen. Indifferent choices were defined as such when the two options were of equal value. RTs for these different types of choices were compared independently of parametric effects using two-tailed independent-samples *t* tests.

Excluding indifferent choices, the first logistic-regression model described accuracy (i.e., the probability of choosing the option with greater value) as a function of the absolute value of the difference between input values and the sum of the input values. The second model included the greater value and the lesser value in their original forms. An analogous pair of complementary linear-regression models was applied to the RT separately for correct and incorrect choices. For the special case of indifferent choices (i.e., difference equals zero), only one model including the sum of values was necessary. As discussed previously, excessively fast contaminant observations were omitted below a lower limit of 300 ms. To facilitate comparison across studies in the meta-analysis, the values were first normalized linearly such that the minimum and maximum values corresponded to zero and unity, respectively. Moreover, parameter estimates for the RT analyses were subsequently normalized such that each regression coefficient was divided by the coefficient for the constant term. To illustrate, a hypothetical coefficient of -0.1 for the greater value’s effect on RT would imply that, ceteris paribus, the RT becomes 10% faster than the mean if the greater value is at its maximum level. Statistical significance was determined for main effects and contrasts using two-tailed one-sample *t* tests and 95% confidence intervals. Despite one-tailed tests being justified by strong a priori hypotheses in most cases, more conservative two-tailed tests were used in their stead here to err on the side of caution. Contrasts of the effects within a regression model were limited to the absolute values of the parameter estimates to avoid redundancy. That is, a significant difference between a signed positive effect and a signed negative effect is less informative than a significant difference between these effects irrespective of sign.

The same analyses of accuracy and RT were employed within each of the other data sets that were included in the meta-analysis. Aggregate results across all data sets were produced by assigning weights to each data set in proportion to the total number of trials included for each analysis.

## Results

### Computational modeling

Multiple theoretically sound hypotheses for competitive interactions have been proposed in the literature—including the absence of any such interactions. Seven models were first assembled a priori per a factorial design (**Fig 2**). Taking into consideration the role of attentional processes, the most successful of these models was then augmented to form an eighth model with superior performance (**Fig 3**). Dissociating and testing specific mechanisms requires a tractable common framework be nested within incrementally varied models representing each potential feature. Thus, the particular versions of the models included in this formal model comparison (**Table 2**) all derived core ideas from published models but were not strictly identical to the original versions.

The race model (**Fig 2A**) [12–15] is the most basic option by virtue of its rigid assumption that the channels representing each option remain independent at all levels. The drift-diffusion model [7,9–11] corresponds to the opposite extreme of a single channel that represents the relative evidence between two inputs collectively. Whereas this work emphasized some degree of neural plausibility, the standard drift-diffusion model is implausible insofar as it simulates only one bidirectional decision signal. In light of this shortcoming, a modified neural drift-diffusion (NDD) model was substituted for its separate decision signals that better align with the arrangement of pathways in the nervous system. This neural implementation still retains the distinguishing feature of sensitivity to differences alone by means of perfect competition between inputs. Such input-dependent competition (**Fig 2B**) could also be imperfect and take the form of lateral inhibition (i.e., input normalization or relative coding) or feedforward inhibition at the level of value-signal inputs, which could entail either subtractive [34,35] or divisive [37–40] transformations of inputs. These two alternatives served as the basis for the subtractive normalization-or-feedforward-inhibition (SNFI) model and its more complex divisive analog, the divisive normalization-or-feedforward-inhibition (DNFI) model. Input normalization and feedforward inhibition are referred to collectively in this particular context because of mimicry in effects at the level of decision signals and thus in ultimate behavioral output. In contrast, state-dependent competition (**Fig 2C**)—that is, competition dependent on the states of accumulating decision signals—can be implemented via downstream lateral inhibition, as for the competing-accumulator (CA) model [26,36]. The hitherto unexplored possibility of input-dependent competition and state-dependent competition coexisting at hierarchical levels (**Fig 2D**) was considered as well with the introduction of a novel pair of hybrids—namely, the subtractive competing-accumulator (SCA) and divisive competing-accumulator (DCA) models.

Despite yielding the best performance among these candidates, the SCA model still failed to account for some qualitative effects in empirical data. This deficiency was addressed as the missing factor of selective attention [32,33] was incorporated into this framework with a parsimonious approximation to produce the supralinear subtractive competing-accumulator (SSCA) model (**Fig 3**).

### Initial model comparison

**Fig 4:**
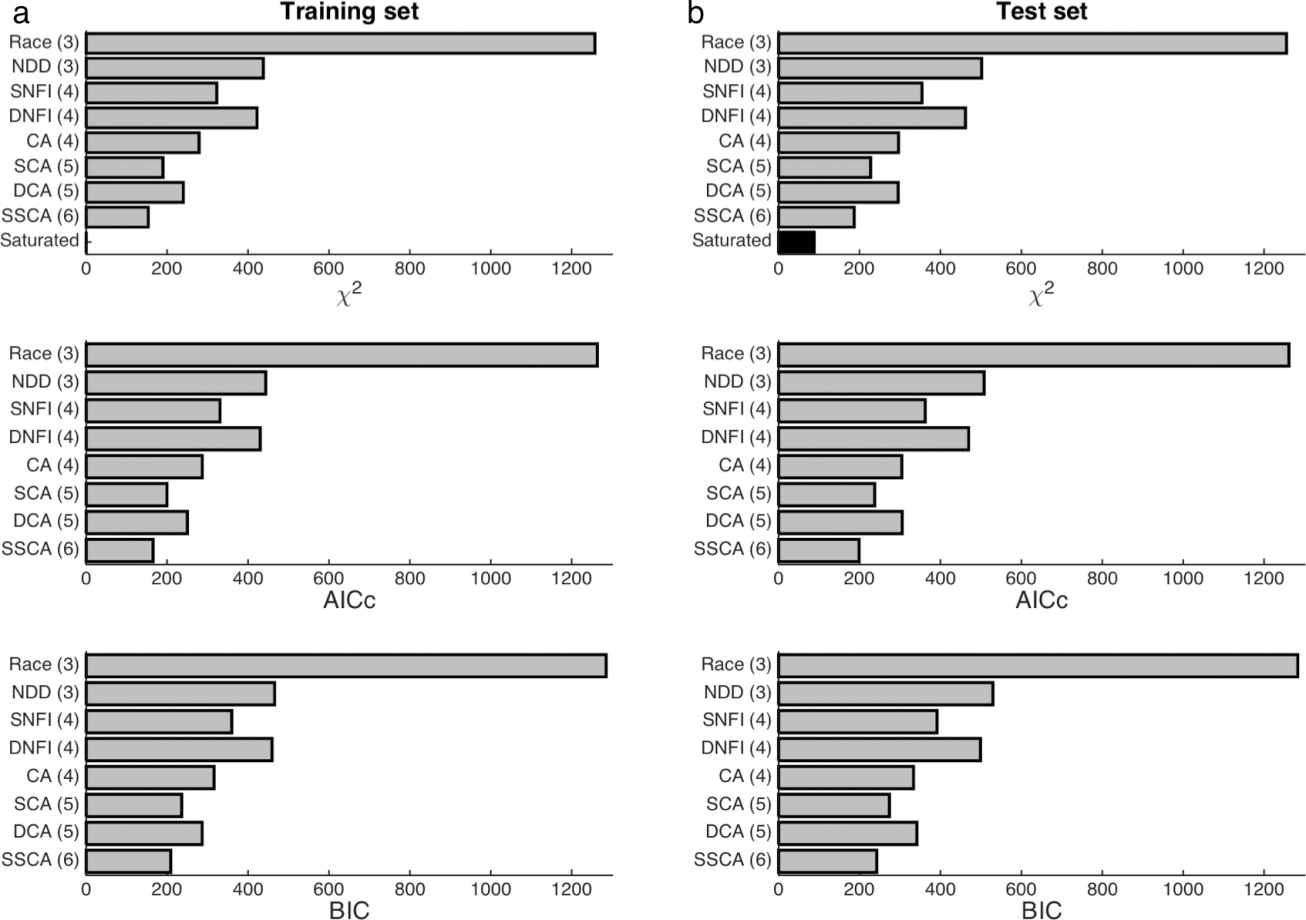
Model comparison. **(a)** The global fitting performance of each candidate model is first shown for the training data set. The χ^*2*^ statistic corresponds to raw lack of fit, but two levels of adjustment for model complexity are also provided in the form of the corrected Akaike information criterion (AICc) and the Bayesian information criterion (BIC). **(b)** A test data set of equal size was reserved for out-of-sample validation. The saturated model revealed the best out-of-sample performance possible with maximal degrees of freedom. Degrees of freedom are listed in parentheses.

As determined by a global metric for goodness of fit to distributions of choices and RTs both within and out of sample, the seven initial models were ranked as follows (in descending order): SCA, DCA, CA, SNFI, DNFI, NDD, race, and null (*p* < 0.05 with the following exception) (**Fig 4**). However, the evidence favoring the DCA model over the CA model was insignificant for the test data set after model complexity was formally taken into account (*p* > 0.05), as could also be demonstrated by the Bayesian information criterion (BIC) [58], which imposes a penalty for each degree of freedom, or even a less stringent alternative in the form of the Akaike information criterion with correction for finite sample size (AICc) [56,57]. Otherwise, additional free parameters were objectively justified, and predictive performance even remained comparable with out-of-sample validation, ruling out overfitting. All fitted parameters, including the baseline input, were robustly nonzero (or greater than unity in the case of attentional modulation) (**Table 3**). In the cases of the hybridized SCA and DCA models, the fitted parameters for input- and state-dependent competition decreased as expected relative to their assignments in the SNFI, DNFI, and CA models, where one level of competition is omitted and so must be compensated for by overfitting at the remaining level. The superior performance of the subtractive models relative to the divisive models was all the more remarkable in light of the greater—albeit unquantifiable—degree of complexity inherent to the divisive models irrespectively of countable degrees of freedom, as this added complexity and nonlinearity would enable more flexible fitting of data in general.

**Table 3:** Fitted parameters.

Model	*b*	*g*	*σ*	*i*_*v*_	*s*	*i*_*d*_	*a*	*χ^2^_Training_*	*χ^2^_Test_*
SSCA	1.434	0.085	2.265	0.465	-	0.0180	1.373	153.26	186.84
SCA	1.195	0.187	2.665	0.470	-	0.0154	-	189.50	227.41
DCA	3.073	5.117	2.571	-	13.80	0.0174	-	240.03	295.48
CA	1.219	0.233	1.933	-	-	0.0252	-	278.85	296.49
SNFI	0.614	0.225	3.968	0.733	-	-	-	322.65	354.44
DNFI	0.109	2.212	3.970	-	1.697	-	-	422.12	461.82
NDD	0.761	0.185	3.803	-	-	-	-	437.77	501.84
Race	0.336	0.233	3.569	-	-	-	-	1257.36	1255.40
Saturated								0.10	87.91
Null								26,606	26,165

The best-fitting sets of parameters for each computational model are listed along with χ^*2*^ statistics. *b* corresponds to baseline input, *g* is gain, *σ* is noise, *i*_*v*_ is value-signal inhibition, *s* is semisaturation, *i*_*d*_ is decision-signal inhibition, and *a* is the exponent representing attentional modulation. The null and saturated models provided extreme lower and upper benchmarks for fitting performance, respectively. As will be the convention for all tables and figures hereafter, the models are listed in descending order of performance.

As the value of one stimulus was not a reliable predictor of the other value, this paradigm’s two-dimensional input space facilitated extraction of effects parametrically related to stimulus values. The subjective value (i.e., utility) of each option was derived from the subject’s linear rating of the desirability of eating the food when presented in isolation. A complete portrait of accuracy and RT was attained by means of two complementary models. One regression analysis included the ranked greater and lesser values individually, and the other featured the absolute difference between the values and their sum, which are orthogonal linear combinations of the original terms. To be thorough, RT was analyzed in this fashion separately for the distinct categories of correct, incorrect, and indifferent choices—with the exception that only the effect of the sum was relevant for indifferent choices. These difference and sum terms can represent (inverse) difficulty and overall motivational (or incentive) salience [59,60], respectively, to an extent, but net effects must be interpreted with prudence because these linear combinations together are sufficiently flexible for mimicry to occur. As an illustration of this caveat, which has been overlooked all too often in previous studies, an effect of the greater value alone could also result in effects of difference (i.e., greater minus lesser) and sum (i.e., greater plus lesser) each with magnitude equal to half of that of the greater-value effect.

**Fig 5:**
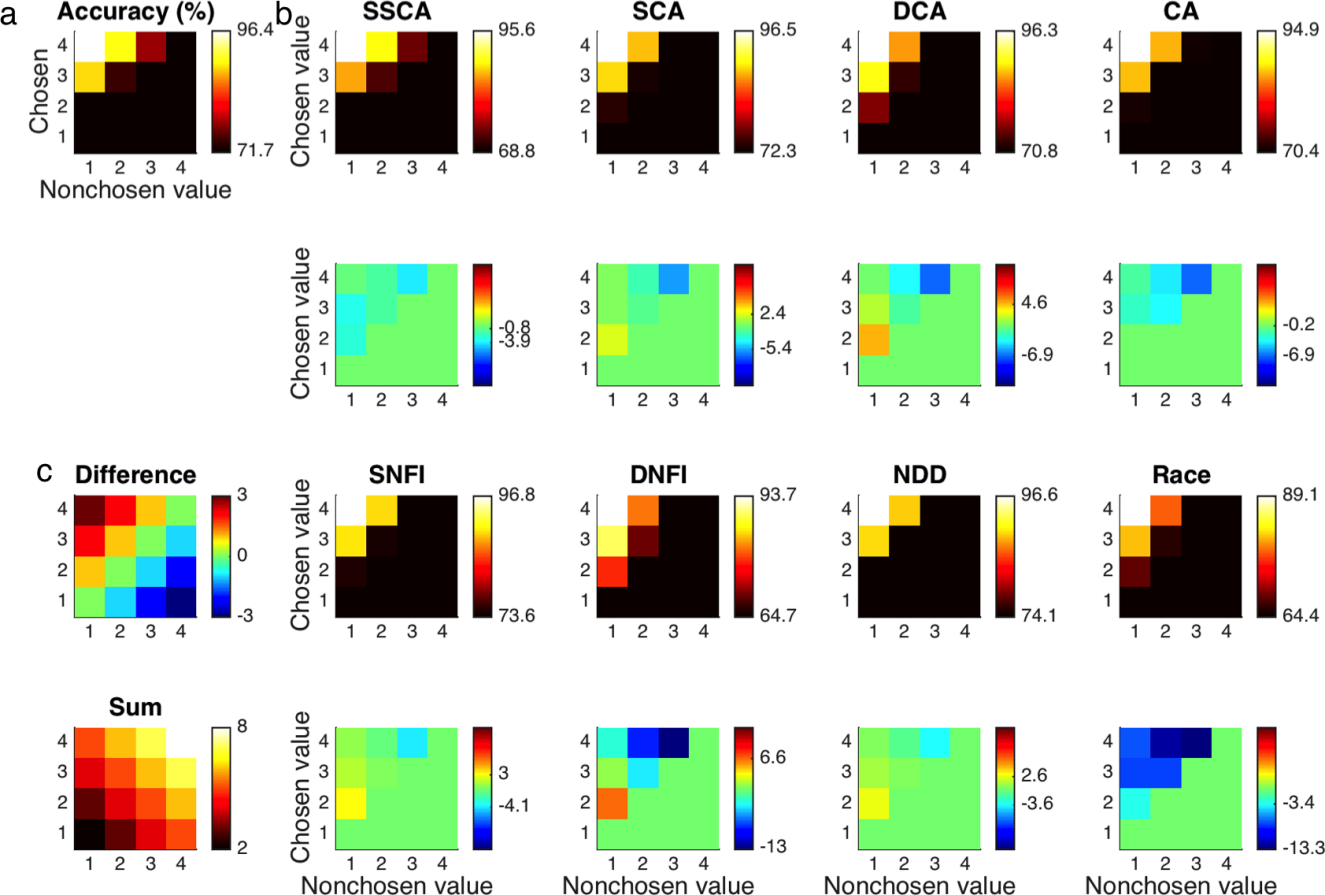
Choice accuracy. **(a)** Choice accuracy (i.e., the probability of correctly choosing the option with greater value) as a function of both values is displayed first for the empirical data set. Only the probabilities of correct choices are provided in the upper-left corners of each panel to avoid redundancy. **(b)** Accuracy is likewise shown for data sets simulated with each of the computational models in the first and third rows. Differences between model predictions and observed results are highlighted in the second and fourth rows. **(c)** The differences between chosen and nonchosen values and their sums are provided for reference.

As expected for the modeled data set, choice accuracy (**Fig 5**, **Table 4**) increased as the greater value increased (*β* = 3.517, *t* = 29.05, *p* < 10^-184^) and conversely decreased as the lesser value increased (*β* = -3.038, *t* = 24.42, *p* < 10^-130^). Notably, the option with the greater value also possessed significantly more weight than its lesser-valued alternative (*M* = 0.479, *p* < 0.05). A corollary of this asymmetry is that accuracy not only increased with the difference between the values (*β* = 3.278, *t* = 29.37, *p* < 10^-188^) but also effectively increased with their sum (*β* = 0.239, *t* = 4.68, *p* < 10^-5^), albeit to a much smaller degree (*M* = 3.038, *p* < 0.05). None of the seven a-priori models were capable of capturing these effects in subjects’ choices—even qualitatively. The NDD model naturally predicted equal weights for the greater and lesser values and missed this pattern of overweighting (*p* >> 0.05), as did the CA model (*p* > 0.05), but the SCA, DCA, SNFI, DNFI, and race models even predicted a contradictory overweighting of the lesser value instead (*p* < 0.05). To clarify, “overweighting” in this context implies deviation from the symmetric weighting of each value prescribed by the normative drift-diffusion model. As detailed below, the SSCA model alone could address this phenomenon.

**Table 4:** Meta-analysis: Choice accuracy.

Data set	Trials	Constant	Greater	|| vs. ||	Lesser	Differ.	|| vs. ||	Sum
JC1	15,600	-0.263*	3.517*	>	-3.038*	3.278*	>	0.239*
		**(0.075)**	**(0.121)**		**(0.124)**	**(0.112)**		**(0.051)**
JC2	6,868	-0.238	3.831*	>	-3.031*	3.431*	>	0.400*
		(0.126)	**(0.199)**		**(0.206)**	**(0.183)**		**(0.087)**
CH	1,128	0.778*	3.211*	n.s.	-3.526*	3.368*	>	-0.158
		**(0.334)**	**(0.599)**		**(0.525)**	**(0.532)**		(0.185)
IK	3,266	0.222*	4.349*	n.s.	-4.154*	4.251*	>	0.097
		**(0.105)**	**(0.364)**		**(0.396)**	**(0.367)**		(0.102)
SL	6,707	0.537*	4.052*	n.s.	-3.650*	3.851*	>	0.201*
		**(0.123)**	**(0.270)**		**(0.280)**	**(0.260)**		**(0.089)**
JL	13,992	0.000	4.768*	>	-3.881*	4.325*	>	0.444*
		(0.107)	**(0.202)**		**(0.196)**	**(0.186)**		**(0.071)**
NS	3,663	-0.158	3.774*	n.s.	-3.236*	3.505*	>	0.269*
		(0.152)	**(0.287)**		**(0.270)**	**(0.269)**		**(0.096)**
Aggregate	51,224	-0.022	4.036*	>	-3.444*	3.740*	>	0.296*
		(0.110)	**(0.193)**		**(0.154)**	**(0.168)**		**(0.049)**
Model		Constant	Greater	|| vs. ||	Lesser	Differ.	|| vs. ||	Sum
SSCA		-0.325*	3.319*	>	-2.890*	3.104*	>	0.214*
		**(0.025)**	**(0.042)**		**(0.043)**	**(0.039)**		**(0.016)**
SCA		-0.035	3.229*	<	-3.373*	3.301*	>	-0.072*
		(0.026)	**(0.045)**		**(0.045)**	**(0.042)**		**(0.016)**
DCA		0.172*	2.990*	<	-3.485*	3.237*	>	-0.248*
		**(0.026)**	**(0.045)**		**(0.044)**	**(0.042)**		**(0.016)**
CA		-0.084*	2.955*	n.s.	-3.005*	2.980*	>	-0.025
		**(0.025)**	**(0.041)**		**(0.041)**	**(0.038)**		(0.016)
SNFI		-0.052*	3.415*	<	-3.514*	3.465*	>	-0.050*
		**(0.027)**	**(0.047)**		**(0.047)**	**(0.044)**		**(0.016)**
DNFI		0.582*	2.096*	<	-3.211*	2.653*	>	-0.558*
		**(0.025)**	**(0.040)**		**(0.039)**	**(0.036)**		**(0.016)**
NDD		-0.053*	3.331*	n.s.	-3.357*	3.344*	>	-0.013
		**(0.026)**	**(0.046)**		**(0.046)**	**(0.043)**		(0.016)
Race		0.127*	1.944*	<	-2.252*	2.098*	>	-0.154*
		**(0.022)**	**(0.034)**		**(0.034)**	**(0.031)**	>	**(0.015)**

Listed for each data set and each computational model fitted to the original JC1 data set are parameter estimates from complementary logistic-regression models of the probability of correctly choosing the option with greater value. The first regression model included the individual greater and lesser values as regressors, whereas the second substituted the absolute difference between values (“Differ.”) as well as their sum. Standard errors of the means are provided in parentheses.

Boldface and an asterisk indicate statistical significance (*p* < 0.05).

Contrasts between absolute values of effects (“|| vs. ||” meaning “absolute value versus absolute value”) are reported with a greater-than sign denoting a greater absolute effect to the left (*p* < 0.05), a less-than sign denoting a greater absolute effect to the right (*p* < 0.05), and “n.s.” (i.e., “not significant”) denoting failure to reject the null hypothesis of no difference between the absolute values of the effects (*p* > 0.05). These conventions apply to all tables hereafter.

**Fig 6:**
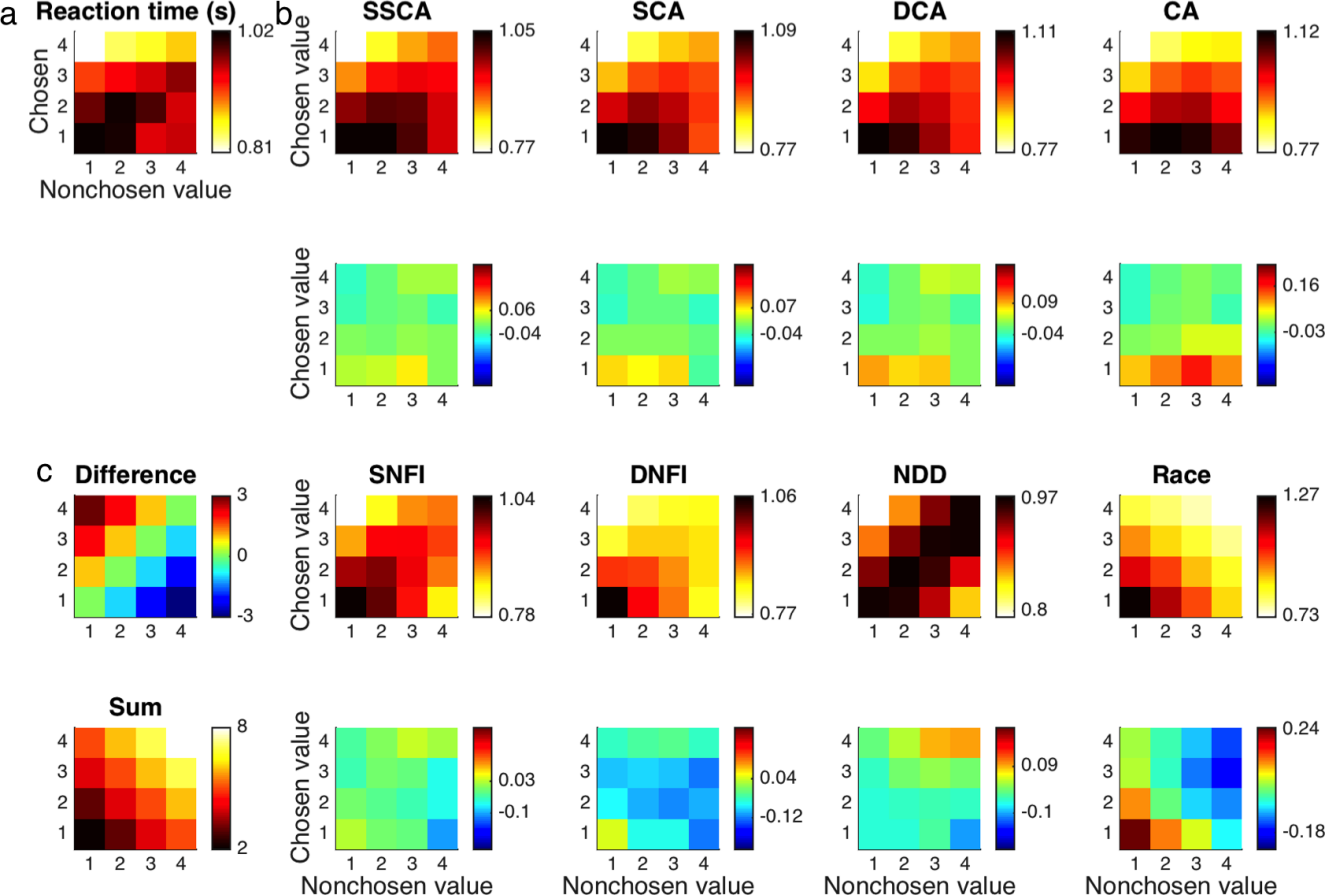
Reaction time. **(a)** Following the conventions of the previous figure, mean reaction time (RT) as a function of both values is displayed first for the empirical data set. **(b)** RT is likewise shown for data sets simulated with each of the computational models in the first and third rows. Differences between model predictions and observed results are highlighted in the second and fourth rows. **(c)** The differences between chosen and nonchosen values and their sums are again provided for reference. The upper-left and lower-right corners of each panel correspond to correct and incorrect choices, respectively, and the diagonal midline between them corresponds to indifferent choices.

When choosing correctly between options of unequal value (upper-left corners in **Fig 6**, **Table 5**), the greater value exerted a speedup effect on RT (*β* = -0.260, *t* = 23.02, *p* << 0.05) while the lesser value exerted a slowdown effect (*β* = 0.066, *t* = 5.73, *p* < 10^-7^). Moreover, the degree to which the greater value sped up the RT exceeded the degree to which the lesser value slowed down the RT (*M* = 0.195, *p* < 0.05). Correspondingly, the RT became faster as both the difference (*β* = -0.163, *t* = 17.43, *p* << 0.05) and the sum (*β* = -0.097, *t* = 15.05, *p* << 0.05) increased, but more so for the difference (*M* = 0.066, *p* < 0.05). All of the more neurally plausible models featuring imperfect competition—namely, the SCA, DCA, CA, SNFI, and DNFI models—could account for this set of effects on RT (*p* < 0.05), whereas the more normative NDD and race models categorically fail to do so regardless of parameter assignments. A byproduct of the NDD model’s assumption of perfect subtractive competition is that the observed effect of sum on RT is missed altogether (*p* >> 0.05) with perfectly anticorrelated weights for the individual values (*p* >> 0.05). The opposite issue applies to the race model due to its lack of competition, such that the weights for the individual values are unequal (*p* < 0.05) but instead both negative (*p* < 0.05) and so produce an effect of the difference weaker than that of the sum (*p* < 0.05). This pattern is to be expected in the presence of “statistical facilitation” [13,61,62] (see Discussion). Such subtleties in effects of individual values on behavior again underscore the importance of taking both inputs into consideration rather than reducing them to a single dimension of difficulty by analyzing on the basis of differences alone, which is standard among previous studies.

**Table 5:** Meta-analysis: Reaction time for correct choices.

Data set	Trials	Constant	Greater	|| vs. ||	Lesser	Differ.	|| vs. ||	Sum
JC1	13,342	1.093	-0.260*	>	0.066*	-0.163*	>	-0.097*
		(0.010)	**(0.011)**		**(0.011)**	**(0.009)**		**(0.007)**
JC2	6,122	1.594	-0.282*	>	0.043*	-0.163*	>	-0.120*
		(0.023)	**(0.017)**		**(0.017)**	**(0.014)**		**(0.010)**
CH	998	1.668	-0.242*	>	0.088*	-0.165*	>	-0.077*
		(0.053)	**(0.043)**		**(0.036)**	**(0.034)**		**(0.020)**
IK	2,562	2.638	-0.742*	n.s.	0.646*	-0.694*	>	-0.048
		(0.079)	**(0.081)**		**(0.092)**	**(0.082)**		(0.029)
SL	6,036	1.480	-0.301*	>	0.197*	-0.249*	>	-0.052*
		(0.017)	**(0.017)**		**(0.018)**	**(0.015)**		**(0.009)**
JL	12,696	1.668	-0.521*	>	0.300*	-0.410*	>	-0.111*
		(0.026)	**(0.022)**		**(0.020)**	**(0.018)**		**(0.010)**
NS	3,041	2.344	-0.320*	>	0.087	-0.204*	n.s.	-0.116*
		(0.161)	**(0.098)**		(0.086)	**(0.080)**		**(0.046)**
Aggregate	44,797	1.563	-0.374*	>	0.182*	-0.278*	>	-0.096*
		(0.159)	**(0.054)**		**(0.058)**	**(0.055)**		**(0.009)**
Model		Constant	Greater	|| vs. ||	Lesser	Differ.	|| vs. ||	Sum
SSCA		1.101	-0.306*	>	0.146*	-0.226*	>	-0.080*
		(0.003)	**(0.004)**		**(0.004)**	**(0.004)**		**(0.002)**
SCA		1.095	-0.299*	>	0.142*	-0.220*	>	-0.079*
		(0.003)	**(0.004)**		**(0.004)**	**(0.003)**		**(0.002)**
DCA		1.093	-0.300*	>	0.169*	-0.235*	>	-0.066*
		(0.003)	**(0.004)**		**(0.004)**	**(0.004)**		**(0.002)**
CA		1.099	-0.303*	>	0.133*	-0.218*	>	-0.085*
		(0.004)	**(0.004)**		**(0.004)**	**(0.004)**		**(0.002)**
SNFI		1.078	-0.278*	>	0.157*	-0.217*	>	-0.060*
		(0.003)	**(0.004)**		**(0.004)**	**(0.003)**		**(0.002)**
DNFI		0.980	-0.221*	>	0.101*	-0.161*	>	-0.060*
		(0.003)	**(0.004)**		**(0.004)**	**(0.003)**		**(0.002)**
NDD		1.009	-0.214*	n.s.	0.212*	-0.213*	>	-0.001
		(0.003)	**(0.004)**		**(0.004)**	**(0.004)**		(0.002)
Race		1.202	-0.314*	>	-0.087*	-0.114*	<	-0.201*
		(0.003)	**(0.003)**		**(0.003)**	**(0.003)**		**(0.002)**

Listed for each data set and each computational model fitted to the original JC1 data set are parameter estimates from complementary linear-regression models of RT in units of seconds for correct choices of the option with greater value that are analogous to the previous logistic-regression models. As in the tables hereafter, these four regression coefficients of interest were normalized with respect to their associated constant term.

Boldface and an asterisk indicate statistical significance (*p* < 0.05).

Incorrect choices of the option with lesser value (lower-right corners in **Fig 6**, **Table 6**) were much less frequent and dominated by pairs of stimuli with small differences in value, resulting in substantially reduced statistical power. Nevertheless, RTs for these enigmatic errors were notably slower than those for correct choices (*M* = 108 ms, *t* = 14.93, *p* << 0.05). All of the models could exhibit this slowing effect to varying degrees (*p* < 0.05). There were also speedup effects of the greater value (*β* = -0.111, *t* = 2.88, *p* = 0.004) and the difference between values (*β* = -0.087, *t* = 2.41, *p* = 0.016), which nearly all of the models shared as well (*p* < 0.05) with the lone exception of a net slowdown effect for the difference in the CA model (*p* < 0.05). Lacking power, however, the absence of significant effects for both the lesser value (*β* = 0.063, *t* = 1.57, *p* = 0.116) and the sum of values (*β* = -0.024, *t* = 1.55, *p* = 0.120) remains ambiguous while at least one of these variables has a significant impact on RT as part of every model’s predictions (*p* < 0.05).

**Table 6:** Meta-analysis: Reaction time for incorrect choices.

Data set	Trials	Constant	Greater	|| vs. ||	Lesser	Differ.	|| vs. ||	Sum
JC1	2,258	1.046	-0.111*	n.s.	0.063	-0.087*	n.s.	-0.024
		(0.024)	**(0.039)**		(0.040)	**(0.036)**		(0.016)
JC2	746	1.559	-0.070	n.s.	-0.009	-0.031	n.s.	-0.040
		(0.067)	(0.071)		(0.073)	(0.066)		(0.030)
CH	130	2.000	-0.153	n.s.	-0.109	-0.022	n.s.	-0.131*
		(0.196)	(0.181)		(0.164)	(0.164)		**(0.055)**
IK	704	2.448	0.023	n.s.	0.169	-0.073	n.s.	0.096
		(0.158)	(0.224)		(0.251)	(0.228)		(0.068)
SL	671	1.498	-0.329*	n.s.	0.394*	-0.361*	>	0.032
		(0.051)	**(0.072)**		**(0.074)**	**(0.068)**		(0.026)
JL	1,296	1.680	-0.421*	n.s.	0.432*	-0.426*	>	0.006
		(0.097)	**(0.111)**		**(0.105)**	**(0.101)**		(0.038)
NS	622	2.808	-0.543*	>	0.185	-0.364*	n.s.	-0.179*
		(0.202)	**(0.142)**		(0.131)	**(0.130)**		**(0.044)**
Aggregate	6,427	1.624	-0.220*	n.s.	0.184*	-0.201*	>	-0.018
		(0.218)	**(0.069)**		**(0.064)**	**(0.061)**		(0.026)
Model		Constant	Greater	|| vs. ||	Lesser	Differ.	|| vs. ||	Sum
SSCA		1.094	-0.114*	>	-0.036*	-0.039*	<	-0.075*
		(0.007)	**(0.012)**		**(0.012)**	**(0.011)**		**(0.004)**
SCA		1.130	-0.162*	>	-0.025*	-0.068*	<	-0.093*
		(0.008)	**(0.012)**		**(0.012)**	**(0.012)**		**(0.004)**
DCA		1.147	-0.159*	>	-0.032*	-0.063*	<	-0.095*
		(0.008)	**(0.013)**		**(0.013)**	**(0.012)**		**(0.004)**
CA		1.164	-0.084*	<	-0.168*	0.042*	<	-0.126*
		(0.008)	**(0.012)**		**(0.012)**	**(0.011)**		**(0.004)**
SNFI		1.073	-0.196*	>	0.074*	-0.135*	>	-0.061*
		(0.007)	**(0.013)**		**(0.013)**	**(0.012)**		**(0.004)**
DNFI		0.998	-0.156*	>	0.007	-0.082*	n.s.	-0.074*
		(0.006)	**(0.009)**		(0.009)	**(0.009)**		**(0.003)**
NDD		1.012	-0.149*	n.s.	0.154*	-0.152*	>	0.003
		(0.007)	**(0.013)**		**(0.013)**	**(0.012)**		(0.004)
Race		1.211	-0.262*	>	-0.150*	-0.056*	<	-0.206*
		(0.005)	**(0.006)**		**(0.006)**	**(0.006)**		**(0.003)**

Listed for each data set and each computational model fitted to the original JC1 data set are parameter estimates from complementary linear-regression models of RT for incorrect choices of the option with lesser value.

Boldface and an asterisk indicate statistical significance (*p* < 0.05).

Decisions made with indifference when the values were matched (diagonals from lower left to upper right in **Fig 6**, **Table 7**) were slower than correct responses as expected with increased difficulty (*M* = 107 ms, *t* = 20.86, *p* << 0.05), which was likewise true of all models (*p* < 0.05). In this case the RT again became faster as the sum of the equal values increased (*β* = -0.069, *t* = 10.76, *p* << 0.05), providing the strongest evidence of an effect of motivational salience. Excluding the NDD model, which cannot account for such an effect outside of the difference under any circumstances (*p* > 0.05), all other models had this prediction in common (*p* < 0.05).

**Table 7:** Meta-analysis: Reaction time for indifferent choices.

Data set	Trials	Constant	Sum
JC1	5,794	1.040	-0.069*
		(0.007)	**(0.006)**
JC2	2,306	1.543	-0.089*
		(0.018)	**(0.010)**
CH	504	1.671	-0.061*
		(0.053)	**(0.023)**
IK	525	2.543	0.006
		(0.133)	(0.069)
SL	1,842	1.549	-0.052*
		(0.023)	**(0.013)**
NS	1,897	2.016	-0.089*
		(0.086)	**(0.035)**
Aggregate	12,868	1.433	-0.070*
		(0.171)	**(0.008)**
Model		Constant	Sum
SSCA		1.058	-0.078*
		(0.002)	**(0.002)**
SCA		1.089	-0.096*
		(0.002)	**(0.002)**
DCA		1.107	-0.097*
		(0.002)	**(0.002)**
CA		1.106	-0.107*
		(0.002)	**(0.002)**
SNFI		1.048	-0.073*
		(0.002)	**(0.002)**
DNFI		1.032	-0.110*
		(0.002)	**(0.001)**
NDD		0.972	-0.001
		(0.002)	(0.002)
Race		1.228	-0.220*
		(0.002)	**(0.001)**

Listed for each data set and each computational model fitted to the original JC1 data set are parameter estimates from a linear-regression model of RT as a function of the sum of values for indifferent choices between options of equal value. The JL data set is not listed here because it does not include indifferent choices.

Boldface and an asterisk indicate statistical significance (*p* < 0.05).

### The SSCA model

**Fig 7:**
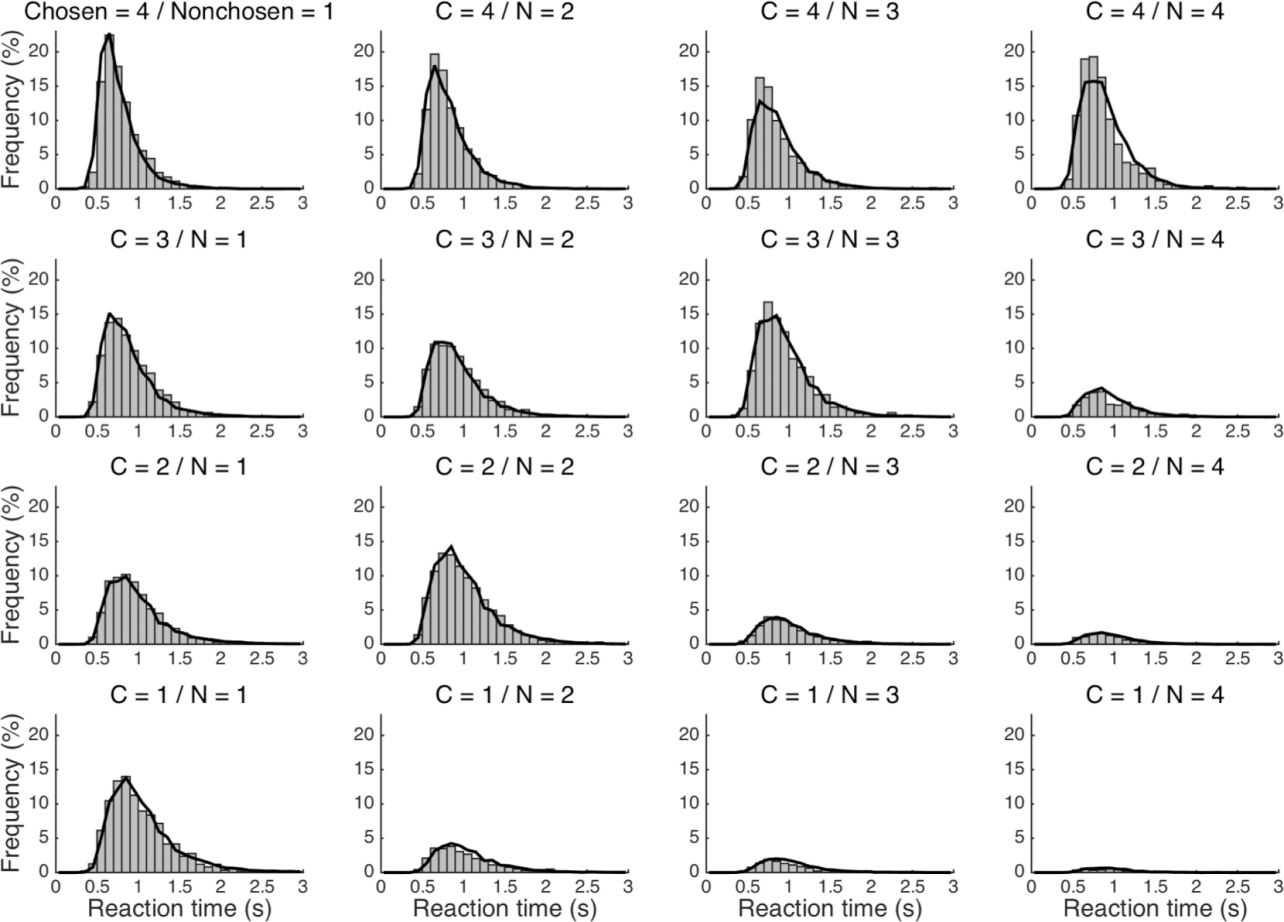
Reaction-time distributions. RT distributions for each combination of chosen (“C”) and nonchosen (“N”) values are displayed with 100-ms bins for the empirical data set (bars) and the data set generated by the preferred SSCA model (lines).

Although the more neurally plausible of the seven a-priori models could account for the more robust impact of a stimulus with greater value on subjects’ RTs, none of these five accounts—to wit, SCA, DCA, CA, SNFI, and DNFI—entailed the analogous overweighting of greater values observed in subjects’ choices. With even the best-performing SCA model still incomplete, its successor, the SSCA model, offered a viable remedy for this deficit with an assumption of attentional modulation, which translates to selective amplification of inputs that are already of high magnitude as part of a positive-feedback loop promoting “winner-take-all” processing [32] (see Discussion). As a static approximation of these dynamics, the impact of attention was parsimoniously reduced to a single free parameter that controls a supralinear power law. This addition enhanced the overall goodness of fit to an extent that justified the extra degree of freedom (*p* < 0.05). Furthermore, the SSCA model demonstrated a qualitative improvement by correctly reproducing the overweighting of options with greater value (*p* < 0.05) as reflected in choices that were similarly characterized by a net positive effect of the sum of values (*p* < 0.05) (**Fig 5**, **Table 4**). With respect to RT, the SSCA model essentially retained all of the aforementioned desirable predictions of the nested SCA model (*p* < 0.05). Despite this qualitative resemblance, however, there was significant quantitative improvement in the correspondence between simulated and actual RT distributions (**Fig 7**).

This data set served as an ideally rigorous test case; that is, the benefits of the SSCA model were even more striking here in light of the fact that central visual fixation was mandatory and sufficient to process the adjacent stimuli simultaneously (**Fig 1A**). It is therefore implied that the downstream effects of covert shifting of the focus of attention could be revealed in the absence of overt eye movements.

### Meta-analysis

To verify the extent to which these findings that were amenable to computational modeling were robust and so would generalize beyond the particular data set under scrutiny, a meta-analysis subsequently tested for qualitative replication of the critical effects with a scope encompassing seven experiments altogether (**Table 1**). In contrast to the modeled data set, which will henceforth be referred to as “JC1,” the added studies featured stimuli that were well separated spatially and thus required saccades in order for each to be foveated (**Fig 1B**). Otherwise, these experimental paradigms generally adhered to the same basic scheme of a 2AFC task for which subjects made preferential choices between randomly sampled foods with uncorrelated subjective values.

With regard to choice accuracy (**Table 4**), the aggregate results of the meta-analysis replicated the findings from the original data set. Across all studies, accuracy increased as the greater value increased (*β* = 4.036, *p* < 0.05) and asymmetrically decreased as the lesser value increased (*β* = -3.444, *p* < 0.05). As before, significant overweighting of the alternative with greater value was apparent (*M* = 0.592, *p* < 0.05). This pattern likewise translated to increasing accuracy as a function of both the difference between the values (*β* = 3.740, *p* < 0.05) and their sum (*β* = 0.296, *p* < 0.05), where the difference had substantially more of an impact (*M* = 3.444, *p* < 0.05). The tendency toward overweighting options with greater value was statistically significant within three data sets (i.e., JC1, JC2, and JL) (*p* < 0.05) and at least trending in the same direction for another three. Likewise, the positive effect of the sum was significant within five data sets (i.e., JC1, JC2, SL, JL, and NS) (*p* < 0.05).

Turning next to RTs for correct choices (**Table 5**), the aggregate results again completely replicated the original set of findings. The greater value made the RT faster across studies (*β* = -0.374, *p* < 0.05), whereas the lesser value slowed it down (*β* = 0.182, *p* < 0.05). There was a similar asymmetry between these oppositional effects (*M* = 0.192, *p* < 0.05). In keeping with that pattern, so too did the RT become faster as both the difference (*β* = -0.278, *p* < 0.05) and the sum (*β* = -0.096, *p* < 0.05) increased with another imbalance between those two effects (*M* = 0.182, *p* < 0.05). All of these relevant trends were fully significant within five data sets (i.e., JC1, JC2, CH, SL, and JL) (*p* < 0.05). Moreover, the remaining two data sets (i.e., IK and NS) were still largely in harmony with the others, such that four of the six critical effects were significant for each (*p* < 0.05).

Whereas the previous results were adequately powered and robust across most of the data sets included in the meta-analysis, the RTs observed for incorrect choices (**Table 6**) were not sampled sufficiently and thus formed less consistent distributions. Despite the additional noise, it remained the case for all studies that incorrect choices tended to be made more slowly than correct choices (*p* < 0.05). Furthermore, the aggregate result suggested that RTs became faster as the difference between values increased for incorrect choices as well (*β* = -0.201, *p* < 0.05). That is, the speedup effect of the greater value (*β* = -0.220, *p* < 0.05) was not significantly different (*M* = 0.036, *p* > 0.05) from the slowing effect of the lesser value (*β* = 0.184, *p* < 0.05). Four data sets (i.e., JC1, SL, JL, and NS) all yielded speedup effects of the greater value (*p* < 0.05) and the difference between values (*p* < 0.05), but only two of these (i.e., SL and JL) also demonstrated a significant slowing effect of the lesser value (*p* < 0.05). Although the NDD model does corroborate such a pattern in error RTs (*p* < 0.05) despite underperforming otherwise, even more data will be necessary to reconcile the discrepancies here and reach more definitive conclusions. For instance, two data sets (i.e., CH and NS) also showed subjects responding more quickly as the sum increased (*p* < 0.05), which is instead in keeping with predictions from the more neurally plausible models (*p* < 0.05).

As concerns the final case of RT for indifferent choices (**Table 7**), which were again delivered more slowly than correct choices across all studies (*p* < 0.05), the aggregate result replicated the speedup effect of the sum of values (*β* = -0.070, *p* < 0.05). Five of the six data sets that included indifferent choices (i.e., JC1, JC2, CH, SL, and NS) exhibited this effect individually (*p* < 0.05).

Altogether, the meta-analysis generally validated the original claims suggested by the modeled data set. Certain qualitative aspects of the findings are summarized in **Table 8**.

**Table 8:** Meta-analysis: Qualitative summary.

Data set	Accuracy						Reaction time												
							Correct						Incorrect						Indif.
	G	v	L	D	v	S	G	v	L	D	v	S	G	v	L	D	v	S	S
JC1 (21)	+	>	-	+	>	+	-	>	+	-	>	-	-	ns	ns	-	ns	ns	-
JC2 (9)	+	>	-	+	>	+	-	>	+	-	>	-	ns	ns	ns	ns	ns	ns	-
CH (2)	+	ns	-	+	>	ns	-	>	+	-	>	-	ns	ns	ns	ns	ns	-	-
IK (4)	+	ns	-	+	>	ns	-	ns	+	-	>	ns	ns	ns	ns	ns	ns	ns	ns
SL (9)	+	ns	-	+	>	+	-	>	+	-	>	-	-	ns	+	-	>	ns	-
JL (14)	+	>	-	+	>	+	-	>	+	-	>	-	-	ns	+	-	>	ns	N/A
NS (6)	+	ns	-	+	>	+	-	>	ns	-	ns	-	-	>	ns	-	ns	-	-
Aggregate	+	>	-	+	>	+	-	>	+	-	>	-	-	ns	+	-	>	ns	-
Model	G	v	L	D	v	S	G	v	L	D	v	S	G	v	L	D	v	S	S
SSCA	+	>	-	+	>	+	-	>	+	-	>	-	-	>	-	-	<	-	-
SCA	+	<	-	+	>	-	-	>	+	-	>	-	-	>	-	-	<	-	-
DCA	+	<	-	+	>	-	-	>	+	-	>	-	-	>	-	-	<	-	-
CA	+	ns	-	+	>	ns	-	>	+	-	>	-	-	<	-	+	<	-	-
SNFI	+	<	-	+	>	-	-	>	+	-	>	-	-	>	+	-	>	-	-
DNFI	+	<	-	+	>	-	-	>	+	-	>	-	-	>	ns	-	ns	-	-
NDD	+	=	-	+	>	0	-	=	+	-	>	0	-	=	+	-	>	0	0
Race	+	<	-	+	>	-	-	>	-	-	<	-	-	>	-	-	<	-	-

This summary reduces the previous four tables to only qualitative assessments of effects on the basis of statistical significance (*p* < 0.05) or lack thereof (*p* > 0.05). Plus signs denote significantly positive effects, whereas minus signs denote significantly negative effects. The NDD model is sufficiently rigid for the null hypothesis to actually be accepted with significance for any effects independent of the difference between values. Approximate trial counts in units of thousands are listed in parentheses for each data set. “G”, “L”, “D”, “S”, and “v” correspond to the headers in previous tables for “Greater,” “Lesser,” “Difference,” “Sum,” and “versus,” respectively. “N/A” stands for “not applicable.”

## Discussion

### Summary

The present study has made strides toward achieving a mechanistic understanding of value-based decision making by formally juxtaposing the explicit predictions of computational models and empirical observations of the behavior of human subjects. The two-dimensional input space common to every experiment tested as part of this meta-analytic approach crucially enabled rigorous assessment of parametric value-related effects. Although the NDD model appreciably outperformed the race model, the strictest normative assumptions of either independent accumulation or perfect subtractive comparison that underlie the race and drift-diffusion algorithms, respectively, were each apparently falsified. By instead representing signals separately but also with imperfect direct competition between them in the form of mutual inhibition, more neurally plausible SSMs offered an account both quantitatively and qualitatively superior while remaining relatively parsimonious. Foremost among these was the SSCA model, a novel connectionist model of a multidimensional nonlinear dynamical system featuring hierarchical levels of competition as well as an approximation of attentional modulation with the efficiency of only six free parameters.

### Optimality or lack thereof

The drift-diffusion model, which is most closely derived from the SPRT, prescribes an optimal solution for the 2AFC paradigm by virtue of attaining the fastest possible mean RT for a given level of accuracy. However, this is but one of many feasible definitions of optimality. The extent to which biology is optimal in domains such as this and which parameters natural selection should optimize remain elusive points of contention [27,63–68]. Whereas Bogacz and colleagues [27] suggested equivalence between the original LCA model [26] and the optimal drift-diffusion model under specific conditions, van Ravenzwaaij and colleagues [67] suggested otherwise and demonstrated that such equivalence only applies under even more extreme conditions that are so improbable and artificial as to be negligible. In a similar vein, the purely descriptive SSCA model is relatively far removed from any provably optimal computations other than the fundamental sequential sampling. Yet, a constrained optimization shaped by evolutionary adaptation need not necessarily align with mathematically provable optimality in a specific context when there also exists demand for versatility across the diverse and dynamic environments that humans and other animals encounter.

The discrepancy between the normative race and drift-diffusion models illustrates one aspect of the nuanced nature of optimality in this context. An oft-cited limitation of the framework shared by the SPRT and the drift-diffusion model is that it does not readily generalize beyond binary decisions as the race model does. The “max-minus-average” variant of the drift-diffusion model directly implied by the standard SPRT is suboptimal [27,69–71], but the unknown optimal standard for multiple alternatives can be approximated asymptotically for sufficiently low error rates by the multihypothesis SPRT [72] and an analogous “max-minus-next” variant of the drift-diffusion model assuming that all signals other than the two with greatest magnitude are somehow filtered out [29,69,71,73]. However, the feasibility of such a scheme when extrapolating to many more than three alternatives has yet to be fully established as tenable. The need to accommodate multiple responses was a relevant factor to motivate laying the groundwork of the race model [74], but it was not the only factor.

Incidentally, Raab [13] was not concerned with matters of optimality and actually first proposed the basic scheme of a race of independent accumulators to account for a documented effect of “statistical facilitation” [61,62]. In the context of a 2AFC paradigm, statistical facilitation implies a tendency towards faster responses as both values increase—that is, not only the value of the better (i.e., more frequently chosen) alternative but also the value of the worse alternative. Under the assumption of independent parallel processes driving each choice, this phenomenon results from additional overlap between each choice’s RT distributions as the accumulation rate of the alternative with lesser value approaches that of the alternative with greater value. The present study made use of these predictions as they starkly contrasted with those of the drift-diffusion and NDD models or more neurally plausible models featuring imperfect competition. The former symmetrically yield slower RTs as the lesser value increases and reduces the relative evidence, whereas the latter for most parameter assignments exhibit a weaker net slowing effect on RT as the lesser value increases but are also flexible enough to accommodate statistical facilitation with a sufficiently low degree of mutual inhibition.

By postulating absolute rather than relative representations of value within independent accumulating signals, the race model can also be regarded as prescriptive or optimal but in a manner altogether separate from the drift-diffusion model. The optimality of the speed-accuracy tradeoff [75] in the SPRT and the drift-diffusion model is predicated on options and sources of evidence for them remaining stable, as is true of most artificial laboratory settings. However, such circumstances are not representative of the dynamic world in which organisms have evolved to make fitness-maximizing decisions in real time that regularly demand flexibility and rapid reaction to changing states [76,77]. Absolute representations of individual stimuli that are insensitive to context could actually be ideal for such situations in which external surroundings and even internal states are unstable. Moreover, ecological validity aside, normative decision theory mandates that, when faced with multiple alternatives, a rational agent whose goal is to maximize utility should make decisions exhibiting “independence of irrelevant alternatives” (IIA) in accordance with the Shepard-Luce choice rule [78,79]. This independence axiom, which entails the probability of choosing one alternative over another being wholly unaffected by any other alternatives, can emerge directly from the race model in the form of a Gibbs softmax function [80,81]. In a certain respect, then, the more neurally plausible SSMs with imperfect competition offer an intermediate alternative that effectively tempers the narrow optimality of the SPRT with the broad optimality of the IIA axiom.

### Features of the SSCA model

The persistent popularity of classical SSMs such as the race and drift-diffusion models among experimentalists also stems from their efficiency and ease of use, and thus even the SSCA model is intended to reach a viable compromise with a minimal increase in complexity outweighed by significant improvement in applicability to actual behavior and neurophysiology. Essentially, the SSCA model has been designed to be somewhat biologically plausible while balancing the constraint of minimizing its parameter count so as to ensure that each element remains fully interpretable and also avoid inappropriate assumptions and overfitting of empirical data. Moreover, fitting the free parameters of a model of this complexity can pose an intractably nonconvex optimization problem with computational demands exacerbated by Monte Carlo simulation of stochastic time series lacking closed-form expressions. Each degree of freedom added intensifies this problem exponentially. In contrast, simpler variants of the race and drift-diffusion models boast more tractable optimization problems further ameliorated by closed-form expressions for distributions of choices and RTs [11,15]. Given these considerations, every free parameter of the SSCA model was carefully selected for proving itself critical both from a theoretical standpoint and from a practical standpoint.

Findings from electrophysiology and other neuroscientific methods at scales ranging from single neurons to whole-brain networks have begun to characterize the dynamics of neural decision-making processes. The SSCA model parsimoniously draws from key neurocomputational principles that have emerged from this line of research. In several regions of the brain, option-selective decision signals encoded in neuronal firing rates have been shown to accumulate up to a threshold level during decision making at a rate proportional to the evidence in favor of a particular option [82–87]. Some additional observations from work in this domain stand out for their core mechanistic implications. Opposing decision signals representing non-preferred alternatives tend to be commensurately suppressed. The rate of accumulation reflects not only stimulus attributes but also the nonspecific urgency to act [87–90]. Thresholds for downstream activation of motor output remain constant [91]. Also relevant is the notion that attending to stimuli or stimulus features—whether perceptual or valence-related—selectively enhances the neural signals representing them [92–98].

Essentially, separate neural ensembles are here assumed to encode option-specific decision signals that compete at hierarchical levels while accumulating activity up to a fixed threshold for motor output at a rate proportional to the value of the option encoded and also boosted by the additional impetus of value-dependent attention and nonspecific urgency signals. Although its influences are broad—also including the feedforward-inhibition model [34,35], the urgency-gating model [99,100], and the drift-diffusion model with attention [33,71]—the SSCA model is distinguished as a member of a narrow class of nonlinear attractor-network models such as the LCA model [26,36] and established biophysical models [31,101,102] that emphasize state-dependent competition via lateral inhibition. However, the SSCA model as a whole is unique and deviates from the original seven-parameter LCA model in multiple ways. In catering to this paradigm, the SSCA model exchanges four free parameters representing leakage, decision-signal thresholds, nondecision time, and starting-point variability for only three new parameters representing baseline input, input-dependent competition, and attentional modulation.

In contrast to the perfect integration of the SSCA model, the LCA model’s assumption that leakage overrides recurrent self-excitation is a strong one and may not apply universally in reality [24,25,103,104]. Indeed, leakage is only an optimal feature for dynamic situations in which information is updated after initial stimulus onset so as to potentially warrant an effective change of mind prior to action. A single free parameter represents the net effect of the balance between leakage and recurrent self-excitation as part of an Ornstein-Uhlenbeck process [105], and this parameter is constrained to be negative (i.e., leakage-dominant) for the LCA model. However, for this particular paradigm where the stimuli predictably remain stable within every trial, there was no compelling evidence of a need for either net leakage or net self-excitation within the framework. Whereas leaky integration is a fundamental characteristic of the dynamics of individual neurons, populations of neurons characterized by a range of intrinsic time constants are nonetheless capable of achieving perfect integration collectively by means of reverberating activity, as is assumed by the SSCA model [106–109].

The decision signal’s threshold for execution is fixed at an arbitrary value to serve as the SSCA model’s scaling parameter. Generally, the interpretation of fitted parameter assignments must be contextualized in the presence of a scaling parameter, which is typical of this variety of models [110]. However, especially with the addition of an urgency signal, a fixed threshold for motor output is actually better justified by observations of neurophysiology [82–84,87–91] than alternative constraints proposed in previous models. As discussed below, the urgency signal can mimic the theoretical collapsing boundary of a diffusion process. Past approaches include fixed within-trial noise [10,17] or—as in the original LCA model—normalized inputs that always sum to a fixed constant [15,36,111]. Tradeoffs are inevitable in this case, but the former solution overlooks the possibility that the fidelity of signaling could vary across conditions being compared. The latter solution, on the other hand, is inflexible in its rescaling of inputs and can degrade both absolute and relative information about their magnitudes.

Decision-making processes are generally expected to be preceded and followed by perceptual stimulus-encoding processes and motoric action-execution processes, respectively, which collectively fall under the concept of nondecision time [10,16]. Whereas these nondecision processes are typically reduced to a single additive constant as part of the estimated RT, such a simplification is prone to miss subtle dynamics of actual neural decision signals [112], which are nonlinear, susceptible to noise, and driven by the urgency to act as well as perhaps attention itself. Furthermore, the ensuing ambiguity surrounding predecision time, postdecision time, and intermittent lapses of attention (e.g., during blinking or saccades) [33,71,113] obfuscates the correspondence between simulated dynamics of neural activity and the time courses of acquired neurophysiological signals. In contrast to fitted nondecision times often in the range of several hundred milliseconds, the initial stages of visual object recognition [114–117], processing of a stimulus’s associated hedonic value [118,119], and response preparation [120] generally begin within 200 ms of the onset of stimulation. Thus, parameterizing the nondecision time not only necessitates an additional degree of freedom that is noisy and particularly susceptible to overfitting but also makes neurally implausible assumptions that cannot be applied directly to computational-model-based analysis of neurophysiological data. The SSCA model instead opts for a biologically constrained predecision time—conservatively set to 150 ms in this value-based paradigm [118,119]—only at the level of value-signal inputs, which are defined with a step function. Downstream decision signals as simulated are never static, evolving explicitly even before the onset of value signals.

Another consequence of the SSCA model’s predecision phase is that starting-point variability emerges from the accumulation of persistent noise before the delayed onset of value-signal inputs. Although this emergent starting-point variability does not have as much flexibility as explicitly parameterized variability in the actual starting point corresponding to trial onset, qualitative effects such as the potential for more frequent fast errors [121] remain without the complications of an additional degree of freedom. Conversely, RT distributions for errors can simultaneously be shifted in the opposite direction relative to correct responses, which typically constitutes the more prominent effect. Along with non-Gaussian noise [122] and asymmetric biases [123,124], across-trial variability in rates of evidence or valence accumulation has been suggested to account for the slower RTs observed for errors [10,15,17,111,121]. Multiple sources of variability across trials as well as hysteresis are entirely feasible insofar as biological signals are inherently probabilistic. Nevertheless, in light of recent reports of neurophysiology reflecting fixed thresholds and urgency signaling, across-trial variability in drift rate may not be the only factor or even a primary factor involved in such discrepancies in timing between correct and incorrect responses [125]. The scope of the present model comparison does not include free parameters for auxiliary sources of variability across trials in the interest of interpretability, but the significance of across-trial variability in starting points, rates of accumulation, onset of input signals, and other parametric elements yet to be explored as part of a more comprehensive model also featuring urgency signals will merit investigation in future research.

Inclusion of a parametric baseline input in the models tested here substantially improves fitting performance but is even more significant for its theoretical implications in relation to signaling of the urgency to act. The stationary threshold of the SPRT is no longer optimal even in the most basic 2AFC paradigm if either of the following commonly occurring conditions apply: the reliability of information could vary from trial to trial, or a cost of effort could be associated with deliberation time within a trial. The psychometric implications of a decaying threshold [126–130], including in particular decreasing accuracy as a function of elapsed time (i.e., slower errors), can bear striking resemblance to those of a nonspecific urgency signal [125]. However, the urgency signal is more neurally plausible when considering the robust evidence of constant thresholds for decision signals as encoded in the firing rates of neurons [82–84,87–91]. This persistent baseline input also prevents decision signals that represent relatively low or even negative (i.e., aversive) values from being deterministically attracted to the null-activity state by the forces of lateral inhibition. Such attraction might also be avoided with the assumption of a sufficiently high starting point for the decision signal at trial onset [67], but the neural plausibility of a nonzero starting point of high relative magnitude remains questionable, which implies yet another free parameter that is ambiguously constrained by neurophysiology.

Whereas the urgency-gating model suggests that a growing urgency signal is multiplicatively combined with a low-pass-filtered evidence signal [90,99,100], the constant baseline input of the SSCA model yields some overlapping predictions for ultimate neural dynamics and behavior by means of a qualitatively distinct mechanism—that is, integration in lieu of independent gating. There is experimental support for the existence of evidence accumulation as opposed to merely urgency accumulation alone, such as the persistent influence of early evidence on decisions when changing information conflicts across different time points within a trial [131–135]. However, inclusion of a low-pass filter with an appropriate time constant can also address these issues to some extent [136]. Further investigation of behavior under deliberately manipulated conditions as well as the flow of information across brain regions at the single-neuron level will prove necessary to fully dissociate urgency gating, the integration of urgency-like inputs, and—albeit to a lesser extent—recurrent self-excitation, which is dependent on the states of decision signals and thus most capable of mimicking nonspecific urgency signals when competing decision signals correspond in magnitude.

Whereas variants of the SNFI, DNFI, and CA models’ schemes for competition have typically each been considered in isolation and even posed as rivals in the literature, the present work has introduced the alternative possibility of complementarity between input-dependent and state-dependent forms of competition. Their synthesis with free parameters for these two levels of competition within a novel hierarchical architecture further distinguishes the SSCA model from the original LCA model, which was instead proposed with the simplest divisive [36] or subtractive [26] input transformations lacking parameterization (i.e., *b* = 0 and *s* = 0 or *i*_*v*_ = 1, respectively). The theoretical interpretation of these rigid transformations was limited to input normalization (or relative coding) alone as opposed to feedforward inhibition. However, although the more fine-grained distinction between lateral and feedforward inhibition may not substantially impact behavioral model predictions at this level of abstraction, this distinction will nonetheless prove relevant for separately identifying value signals and decision-making signals in the brain, where putative roles of different inhibitory mechanisms can be tested for directly. This nonparametric divisive normalization also has been put forth in part to eliminate the aforementioned scaling problem and reduce the number of free parameters, but that solution is less plausible than the one proposed herein. The present results instead suggest the need for the flexibility of parameterized input-dependent competition in a descriptive model even when including state-dependent competition despite the cost of the added complexity. For example, the speedup effect of the sum of values on RT is missed with nonparametric subtraction, and with nonparametric division this effect of sum is too strong relative to the effect of the difference between values even to the point of outweighing the latter, contrary to what is observed in behavior.

Selective attentional modulation of value signals and in particular the asymmetry of its allocation in proportion to value was demonstrated to provide a viable account for the overweighting of greater values observed in choice data as discussed previously. Although at first drawn to perceptually salient [137] or novel [138] stimuli [139], attention disproportionately amplifies value signals of greater magnitude as they are integrated into respective decision signals because more attention also tends to be allocated for more rewarding options—and particularly so in the final moments prior to making a decision when acquisition of necessary information approaches its saturation point [32,33,71,73,113,140–142]. Reflecting preferential looking [143] and the mere-exposure effect [144] in parallel with information seeking, this cascade effect of gaze and attention more generally in response to motivational salience [60] or incentive salience [59] emerges as a positive-feedback loop biasing decisions. Of additional note is that these effects were even present as a reflection of covert shifting of the focus of visual attention in the absence of eye movements for the modeled data set.

Whereas Stevens’s power law [145] from psychophysics in the vein of a nonlinear transfer function could in principle accommodate the possibility of supralinear as well as sublinear input-output relationships, such an interpretation is not merited here because the subjective perception of hedonic value constitutes a special case that is described by a sublinear function in accordance with Gossen’s law of diminishing marginal utility from classical economics [146,147]. Supralinear manifestations of Stevens’s power law in general may actually themselves be a manifestation of the “winner-take-all” attentional phenomenon in question to some extent because attention permeates even processes at levels of representation independent of overt motoric orienting. Moreover, ratings of subjective value were already explicitly mapped onto a linear scale here. Linear rating scales are ubiquitous outside the laboratory and quite familiar for these human subjects, and such linearized subjective ratings have been shown to be linearly related [148] to fully incentive-compatible [47] measurements of one’s “willingness to pay” for an item with currency [149]. Thus, it may be the case that, over time, the positive-feedback loop emerging from attentional modulation during comparison that is essentially averaged out in the present model can effectively override the initial scaling of subjective value as can be observed in independent evaluations of isolated stimuli.

Emphasizing net effects, the static power-law implementation of attention currently used in the SSCA model is only intended to suffice as the most parsimonious solution to the challenging problem posed by the role of attention, however. At this early stage, forcing potentially impactful mechanistic assumptions about the precise nature of attentional processes would not be appropriate in consideration of the fact that they still remain poorly understood in the context of decision-making processes. Further investigation of the neural mechanisms underlying such attention and their temporal properties will be necessary. For example, findings suggesting that attention improves signal-to-noise ratios not only via amplification of gain [92–94, 96–98] but also via reduction of noise [95] or converse suppression of unattended input [150,151] have important implications for modeling. An enhancement of signal-to-noise ratio is consistent with evidence that visual fixations at the beginning of a trial tend to be directed at stimuli from which information must be obtained in contrast to fixations toward the end of a trial that tend to be directed at more rewarding stimuli [142] and thus asymmetrically drive the positive-feedback loops formed across at least attentional and value-encoding signals if not also decision-making signals. Moreover, in addition to this more top-down motivational salience, bottom-up perceptual salience directly tied to physical characteristics has the potential to initially exert a stronger influence on the attraction of attention to particular stimuli under consideration [137], producing biases even in contexts where only hedonic value is relevant [73,152].

For future investigation, the spatial focus of attention can be approximated with high temporal resolution by measuring the direction of eye gaze as it shifts within a trial as part of eye-tracking studies. Along with neurophysiological measurements, eye tracking will prove fruitful for this line of research because it can be used to empirically test more complex models with an aim to describe not only how attention and visual fixation shapes decision-making processes [33,71,113] but also how eye movements are generated as part of this [73]. That is, attentional processes themselves can be modeled beyond their net effects as yet another dynamical system embedded within this framework. On the other hand, the scope of the present work as an initial step is structured so as to demonstrate in a generalizable manner the effectiveness of these neurally inspired tools even when only choice and RT data are available, which is typically the case for empirical computational studies of this nature.

Finally, as the SSCA model aims to an extent for a descriptive and neurally plausible account, it forgoes the simplification of ballistic accumulation—that is, deterministic accumulation in the absence of within-trial noise—which has been proposed for tractability and easier fitting of empirical data [15,111,153–155]. Although ballistic accumulation does offer practical advantages, this feature would fundamentally alter the chaotic and nonlinear dynamics of the model, resulting in overly rigid “winner-take-all” attractor effects. The same is true of the model’s psychological interpretation inasmuch as the algorithm would no longer correspond to a sequential-sampling process, which is necessarily stochastic. The intrinsic stochasticity of biology strongly supports the notion of decision making as sequential sampling rather than ballistic accumulation, however.

### Levels of analysis in computational modeling

Opting for yet more detail than connectionist models such as the SSCA model, biophysical models such as that of Wang [31] can grow substantially more complex but nonetheless preserve the fundamental structure proposed herein. As a testament to this high-level similarity, the schematic of the mean-field reduction of the biophysical model [101,102] generally aligns with that of the CA model depicted in **Fig 2C** [27]. Reducing a population of neurons with correlated dynamics to a collective unit has indeed been shown to be a valid simplification [156,157]. The SSCA model and certain variants of the LCA model potentially provide a more parsimonious account for certain empirical findings that this biophysical model has been put forth to explain, including the prominent effects of the sum of values and the difference in values on RT and aggregate neural activity [158], the relationship between the balance of neural excitation and inhibition and the speed-accuracy tradeoff [159], and a positive correlation between the bias in favor of choosing alternatives with greatest value and the values of alternatives with least value when more than two are under consideration [160]. Nevertheless, there is no “correct” degree of abstraction for modeling phenomena of the brain and mind; models at levels of analysis even as seemingly disparate as biophysics and cognition should be regarded as complementary and ultimately linkable rather than in rivalry [30].

In contrast with such biophysical models, the relative strength of the low-dimensional SSCA model is endowed by its parsimony, interpretability, and generalizability. Tests of data from an independent hold-out sample verified that overfitting was not of concern for the SSCA model, which is a critical feature. Aside from the obvious advantage of mitigated computational demands, low dimensionality is especially relevant for situations in which a model must be fitted to multiple data sets while remaining valid and meaningful for comparison across data sets and with alternative models. Generalization across experimental settings with varied tasks and temporal properties warrants freedom in the assignment of tuning parameters, which the biophysical model lacks in the ambiguity surrounding its degrees of freedom. That is, the parameters of the biophysical model are fixed by default and necessarily derived from past experimental measurements made in particular parts of the brain in a single species while engaged in a single task—for example, lateral intraparietal cortex (i.e., “area LIP”) in a rhesus macaque while performing a random-dot-motion task with saccades [31]. However, considering that the predictions of more complex models correspondingly depend even more heavily on their parameter assignments as well as the parameters of the task, a valid model comparison requires that all relevant parameters of any model under consideration be optimized for the training data in order to ascertain each candidate’s true potential.

The models in this study are nested within a common neural-network framework and distinguished by isolated key features for the sake of commensurability. Comparing models that differ in complex ways can prove futile to the extent that interpreting the exact sources of unique predictions is limited by contamination from other sources. Thus, any extensions of the SSCA model, which is minimalistic by design, should be constructed with one incremental change at a time and tested for qualitative more so than quantitative improvement at describing empirical data in order to justify every additional assumption and the ensuing obstacles posed by fitting and theoretical interpretation [161]. Constraining models to be as simple and parsimonious as possible is advantageous for testing the consequences of incremental changes to enable concrete understanding of fundamental mechanisms. Basic models should be augmented to make them more neurally plausible from a theoretical standpoint, but accounting for effects related to stimulus attributes in empirical data remains the foremost priority. For instance, the race model is fully nested within the SNFI and CA models by assuming no competition with *i*_*v*_ = 0 and *i*_*d*_ = 0, respectively, and effectively nested within the DNFI model if semisaturation is sufficiently greater than input magnitudes (i.e., *s* >> *Σ*_*x*_*V*_*x*_). The NDD model, on the other hand, is only nested within the SNFI model with *i*_*v*_ = 1. The SNFI and CA models are in turn nested within the SCA model, whereas the DNFI and CA models are nested within the DCA model. The additional free parameters could be adequately justified only with a demonstration of objectively superior performance in fitting empirical data.

This incremental “top-down” approach to modeling based on measurable functional properties stands as a viable alternative to the massively parallel “bottom-up” approach advocated in using biophysical models, which instead impose many strong but putatively biologically grounded assumptions at once to generate complex emergent phenomena. Although undoubtedly more applicable at the single-neuron level, the bottom-up approach can be hampered by issues related to high dimensionality, lack of interpretability, the potential for impactful inappropriate assumptions, questionable generalizability, ambiguity in selection of tuning parameters, and the risk of overfitting if tuning parameters are introduced. In addition to the aspect of model complexity quantified with statistical criteria that reflect explicit degrees of freedom, there is an unquantifiable aspect implicit in the model’s ostensible physical implementation. As a case in point, a neural implementation of a divisive transformation of input would entail stricter structural assumptions than a less complex subtractive transformation despite both types similarly being reducible to only one additional free parameter here. If the juxtaposition of the state- and input-dependent competition of the CA and SNFI or DNFI models, respectively, were transposed from the connectionist framework to a biophysical framework, compound interactions among the many elements of such a detailed system, which are not completely understood and also highly dependent upon context and parameter assignments, would severely limit inference with regard to the mechanistic implications of any disparities.

Even without a foray into the most elaborate biophysics, one could hypothesize a connectionist model still more neurally plausible than the SSCA model by incorporating elements as varied as increased connectivity with both excitatory and inhibitory feedback connections, value and execution signals with more complex dynamics than step functions [162], noise specific to distinct layers of neural ensembles or subprocesses, state-dependent (e.g., mean-scaled) sources of within-trial noise [28,39,163,164], and across-trial variability as discussed earlier. However, selecting a model with so many features to relate to empirical data can quickly grow into an intractable problem in the presence of complex nonlinear interactions that prevent dissociating and fitting the relevant parameters so as to discern among the myriad of possible combinations. Many degrees of freedom, reciprocal connections, the associated feedforward and feedback loops, and partially redundant mechanisms in a complex dynamical system can give rise to functional mimicry and thus overlapping predictions for output that further limit interpretability. Furthermore, if parameter optimization is successful, the addition of any free parameter within reason is likely to at least marginally improve the quantitative fit of a model merely by virtue of an added opportunity for nonlinearity. A challenge for future work thus arises in assigning priority to certain elements over others while it is impractical to simply include every element that can be theorized in a model. Incremental augmentations of the model could then be achieved by deliberately controlled experiments that would yield testable predictions contingent on inclusion of a given element that in theory better emulates actual nervous systems at a more abstract computational level.

### Computational-model-based analysis of neurophysiological data

One of the principal goals of computational cognitive neuroscience [165] is to formulate generative models that encompass brain, mind, and behavior together. To this end, a hybrid SSM such as the SSCA model that has been honed to balance the demands of efficiency in modeling and representativeness of neurobiology can also cater to computational-model-based analysis for neurophysiological data [44,45]. That is, the SSCA model can ultimately be related to not only behavioral output but also neural activity such as blood-oxygen-level-dependent (BOLD) signals from functional magnetic-resonance imaging (fMRI) with its high spatial resolution (e.g., [166]) or event-related potentials from electroencephalography (EEG) with its high temporal resolution (e.g., [20]). Attempts have been made to relate output of normative SSMs such as the race and drift-diffusion models to neurophysiological data under the assumption of adequately representing the brain’s functional architecture, but the SSCA algorithm could be appreciably more effective in such endeavors with the benefit of greater neural plausibility, better fits of behavior, and nonlinear flexibility. For any given trial, this model can generate temporally precise predictions for aggregate neural activity from stimulus onset to the time of response as collectively determined by attributes of all stimuli, the subject’s choice, and the RT. Such comprehensiveness is critical and actually sets the approach proposed herein apart from previous neuroimaging studies’ attempts to identify decision-making processes with computational models instead limited to some subset of that information available to describe the input and output of individual trials.

In terms of accuracy and interpretability, this fully model-based approach to localization of decision-making processes in the brain has far more potential than conventional methods that instead often rely on a functional signature involving reduction of the information in each trial to the relative evidence between options as a proxy for normative difficulty. These linear signatures generally take the form of either the absolute difference between the values of options or the signed difference between chosen and nonchosen values, but the latter formulation cannot even be reconciled with speedup effects of RT and concomitant negative effects on cumulative neural activity as a function of the absolute difference for incorrect as well as correct choices. Although the RT is potentially a superior alternative for its direct reflection of actual behavior rather than parameters of stimuli, it is nonetheless also insufficient as an independent variable for the brain not only because of omission of information about choices and inputs but also because of further nonlinearity in the relationship between RT and the underlying neural dynamics that can be simulated on a trialwise basis.

For each condition under which they are engaged, neural decision-making processes should exhibit correlation between observed signals and the simulated signals of the SSCA model to the extent that these simulations would be derived from a theoretically sound and neurally plausible model empirically proven to fit well. Decision-making processes can thus be identified selectively among all processes active in the brain during a given task, including but not limited to the value-encoding and action-execution processes also within the scope of the model. Specificity or lack thereof to experimentally manipulated conditions can then be determined. This methodology enables principled “forward inference” across various conditions of interest by revealing qualitative dissociations in recruitment of particular brain areas during decision making [167,168]. The precision afforded by a comprehensive yet tractable account of both the brain and behavior in terms of explicit computations and algorithms will prove pivotal in achieving a complete mechanistic understanding of decision making across diverse settings.

## Supporting information

S1 File Data. All data sets are included.
(ZIP)
