# Correction: Value-based decision making via sequential sampling with hierarchical competition and attentional modulation

**DOI:** 10.1371/journal.pone.0203093

**Published:** 2018-08-23

**Authors:** Jaron T. Colas

In the Materials and methods section, there is an error in the second equation under the section titled “Computational modeling.” Please view the complete, correct equation here:

$$\forall \;x:\;v_{x}\left(t\right)=\{\begin{array}{ll} 0, & t<T_{0}\\ V_{x}, & t\geq T_{0} \end{array}$$

In the fourth paragraph of the section titled “Computational modeling,” there is an error in the penultimate sentence; the term should read “*N(0*,*σ*^*2*^*)*_*x*_*(t)*” instead of “*N(0σ*,^*2*^*)*_*x*_*(t)*”. The correct sentence is: The final term, *ε*_*x*_*(t)* (or *N(0*,*σ*^*2*^*)*_*x*_*(t)* henceforth to be explicit), combines all sources of noise into a Gaussian distribution with mean *μ* = 0 and parameterized standard deviation *σ* that is drawn from independently within each alternative’s subsystem at every time step.

In the Materials and methods section, there is an error in the equation under the section titled “The supralinear subtractive competing-accumulator (SSCA) model.” Please view the complete, correct equation here:

$$\forall \;x:\;v_{x}\left(t\right)=\{\begin{array}{ll} 0, & t<T_{0}\\ V_{x}^{a}, & t\geq T_{0} \end{array}$$
